# Bioinspired Strong and Tough Layered Bulk Composites via Mycelial Interface Anchoring Strategy

**DOI:** 10.1002/advs.202413226

**Published:** 2025-03-24

**Authors:** Hao Wang, Jurui Liu, Zhangyu Wu, Xianfeng Chen, Kai Jin, Jie Tao, Bin Wang

**Affiliations:** ^1^ Department of Mechanical Engineering City University of Hong Kong 83 Tat Chee Avenue Kowloon Hong Kong China; ^2^ School of Materials Science and Engineering Southeast University Nanjing 211189 China; ^3^ A*STAR Quantum Innovation Centre (Q.InC) Institute for Materials Research and Engineering (IMRE) Agency for Science Technology and Research(A*STAR) Singapore 138635 Singapore; ^4^ School of Materials Science and Engineering Ocean University of China Qingdao 266402 China; ^5^ College of Materials Science and Technology Nanjing University of Aeronautics and Astronautics Nanjing 211106 China

**Keywords:** bioinspired structural composites, hyphae, interface anchoring, layered bulk composites

## Abstract

Lightweight structural composite materials are widely used in automobiles, aerospace, and other fields. However, achieving the integration of structural and functional properties, such as the ability to monitor external forces, remains a significant challenge. Nacre and turtle shells in nature are strong and tough due to their unique ordered structure of alternating soft and hard phases. Inspired by this, an interface anchoring strategy is proposed which leverages hyphae (filamentous structure forming the vegetative part of fungi) to fix the hard‐phase graphene nanosheets (GNs) and the soft‐phase intertwined polymer matrix to form theree‐dimentional (3D) layered bulk composites (LBCs). The growth pattern of fungi is utilized to place GNs and assemble polyethylene glycol‐polyvinyl alcohol (PEG‐PVA) to fabricate the LBCs, which is different from most existing preparation methods of bulk biomimetic composites. The LBCs exhibit self‐regenerative capabilities and are amenable to scalable manufacturing. These composites demonstrate impressive mechanical properties, including a specific strength of 92.8 MPa g cm^−3^, fracture toughness of 6.5 MPa m^−1/2^, and impact resistance of ∼3.1 kJ m^−2^, outperforming both natural nacre and other biomimetic layered composites. Furthermore, the LBCs display effective protective warning functions under external force stimulations, making them a promising material for anti‐collision applications in industries such as sports and aerospace.

## Graphical abstract

An interface anchoring strategy, which fixes the interface between the soft and hard phases to immobilize 2D materials, by leveraging the growth of biological living mycelium is proposed. This approach results in the formation of a lightweight and strong composite material (the LBCs) with a layered structure composed of soft and hard phases.

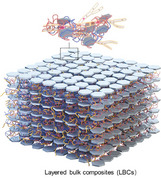

## 1 Introduction

Lightweight structural materials are crucial in aerospace, transportation, and construction engineering applications.^[^ ^1^, ^2^, ^3^ ^]^ There has been an increasing demand for materials that are strong, tough, and capable of self‐monitoring under external loads in fields like sports and aerospace. Self‐monitoring soft materials have been reported in the literature,^[^ ^4^, ^5^, ^6^, ^7^ ^]^ such as conductive fibers^[^ ^4^ ^]^ and gels,^[^ ^5^, ^6^, ^7^ ^]^ while there are few tough and lightweight structural materials with self‐monitoring properties.

Natural materials, such as nacre and turtle shells, exhibit exceptional strength and toughness due to their three‐dimentional (3D) hierarchically ordered stacking structures and multi‐scale interfaces.^[^ ^8^, ^9^, ^10^, ^11^ ^]^ These natural formations have inspired the design of materials with high mechanical properties.^[^ ^3^, ^7^, ^12^, ^13^, ^14^, ^15^, ^16^ ^]^ Graphene, with a breaking strength of 130 GPa and a Young's modulus of 1.0 TPa, has garnered significant attention as an ideal reinforcing material.^[^ ^17^ ^]^ However, these exceptional mechanical properties are observed at the nanoscale and have not yet been achieved at the macroscale. There have been studies focused on organizing graphene into layered materials, demonstrating the effectiveness of these methods in creating materials with impressive layered structures and mechanical properties.^[^ ^18^, ^19^, ^20^ ^]^ For instance, Wan et al.^[^ ^18^ ^]^ obtain high‐strength scalable graphene sheets through freeze‐stretch‐induced alignment, enhancing the isotropic in‐plane sheet strength to 1.55 GPa while maintaining high Young's modulus, conductivity, and weight‐normalized shielding efficiency. Li et al.^[^ ^19^, ^20^ ^]^ introduce a continuous plasticizing stretching approach to adjust the spontaneous wrinkles of graphene sheets into a crystalline order, producing continuous graphene paper with a Hermanns order as high as 0.93. This crystalline graphene paper exhibits excellent mechanical properties, with a tensile strength of 1.1 GPa and stiffness of 62.8 GPa, along with high electrical conductivity. In addition to these techniques primarily focusing on producing 2D films or 1D fibers, directly creating 3D bulk materials is important to wider engineering applications. Several reported approaches, e.g., roll‐pressing methods^[^ ^20^, ^21^ ^]^ exemplified by Kim et al.,^[^ ^21^ ^]^ have prepared graphene‐PMMA laminates that show promise in fabricating 3D layered bulk materials. With these methods offering certain advantages, realizing a good microstructure control for high‐performance 3D layered bulk materials remains challenging. This microstructure control is key to effective stress transfer, high mechanical properties,^[^ ^8^, ^22^ ^]^ and enhanced self‐sensing capabilities in lightweight applications.

The interface between different phases (a major microstructure feature) in biomimetic lamellar structured composites plays a crucial role in stress transfer and mechanical performance. Researchers have made significant efforts to achieve optimal interfaces through various strategies,^[^ ^22^, ^23^, ^24^, ^25^ ^]^ such as designing polymer molecular interactions including H‐bond interactions,^[^ ^22^ ^]^ electrostatic attraction,^[^ ^23^ ^]^ and network entanglement,^[^ ^24^ ^]^ and tuning the thickness and properties of polymer interlayers.^[^ ^25^ ^]^ These strategies have advanced the fabrication of biomimetic composites with main focuses on designing chemical reactions and/or engineering methods while optimizing the interface through direct biological approaches for microstructure control is under development. Addressing this is essential to integrating the structural and functional properties simultaneously within a single composite material.

**Figure 1:**
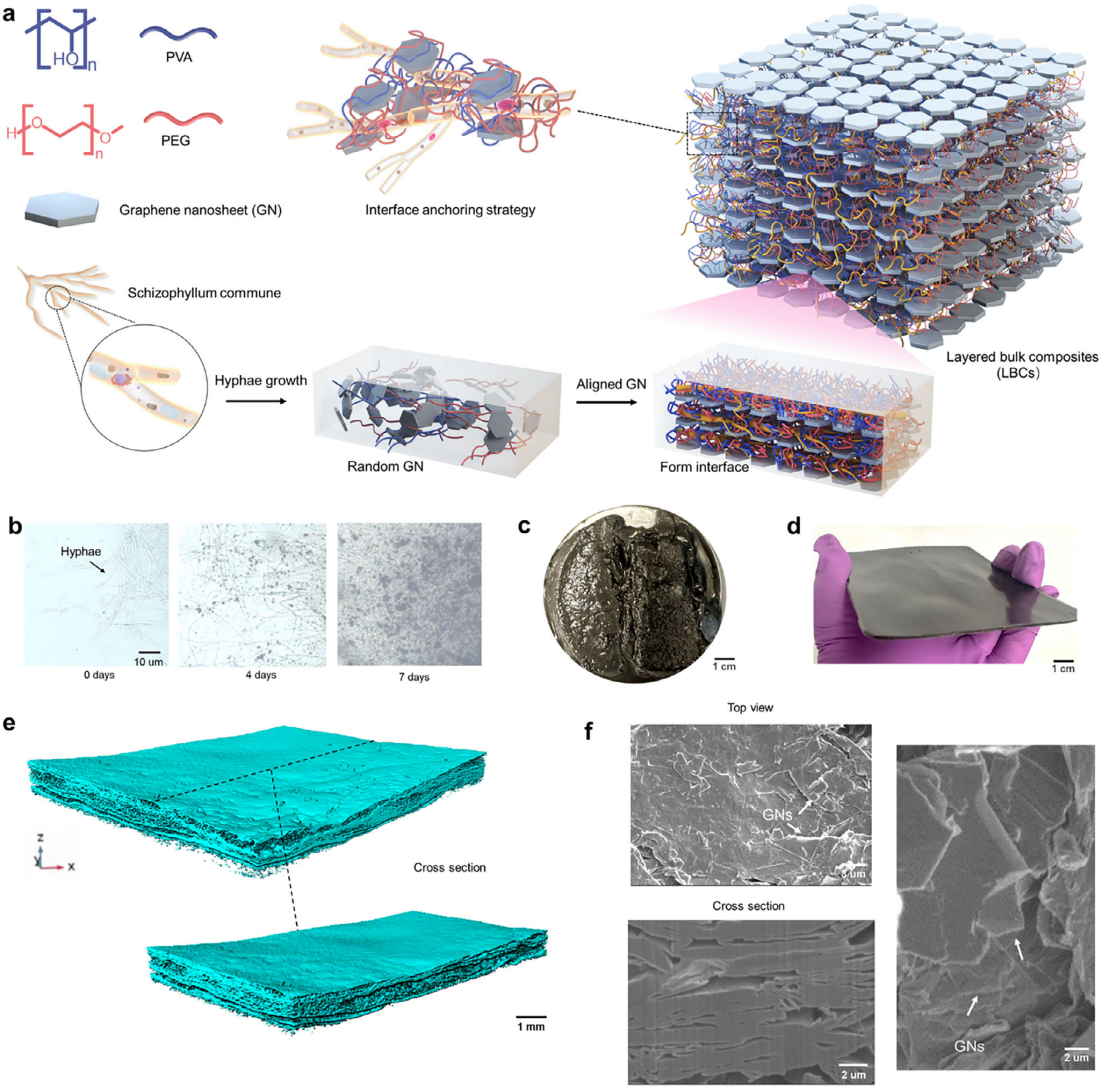
The fabrication for layered bulk composites (LBCs). a)The schematic illustration of the design and fabrication process for the LBCs. The interface anchoring involves selecting the entangled PVA and PEG chains as the soft phase, and the GNs as the hard phase. The GNs are positioned through the growth of *Schizophyllum commune* in the solution and induced the entangled PVA‐PEG chains wrapping the hyphae surface. This process allows the hyphae anchored interface formed via PVA‐PEG‐GNs wrapped by the hyphae. b) Optical microscopic images. Living *Schizophyllum commune* fungi are introduced into the PVA‐PEG solution with 15% GNs, and grown for 4 days, 7 days. Digital camera photos: c) After 10 days of growth. d) After the drying and compression processing. e) 3D CT images. f. SEM images from the top surface, section, and fracture surface.

In this work, we introduce an interface anchoring strategy by fungal mycelium to fabricate scalable 3D bulk composites inspired by the alternating soft and hard phases structures found in natural nacreous materials. The design for layered bulk composites (LBCs) is schematically depicted in **Figure** **1**a. Hyphae, which grow from the germination of fungal spores, have the unique ability to adjust their growth patterns based on local nutritional conditions. They rapidly expand at their tips and exhibit branching, turning, and occasional fusion, resulting in the formation of a complex 3D, fibrous mycelial network.^[^ ^26^, ^27^, ^28^, ^29^ ^]^ Moreover, the fungi species of *Schizophyllum commune* can assemble water‐soluble polymer chains in solutions, facilitating the formation of an interconnected network through hydrogen bonding, which contributes to the material's enhanced compressive strength.^[^ ^30^ ^]^ High molecular weight polyvinyl alcohol (PVA) and polyethylene glycol (PEG) can be entangled with each other via hydrogen bond interactions in aqueous solutions, exhibiting excellent elasticity and toughness,^[^ ^31^, ^32^ ^]^ making them suitable as the soft phase in biomimetic composites. Graphene nanosheet (GN)^[^ ^17^ ^]^ acts as the hard phase. The LBCs are prepared by leveraging the growth of *Schizophyllum commune* mycelium which assembles PVA‐PEG entanglements to wrap GNs on the hyphal surface, followed by removing water and applying pressure. This strategy utilizes the growth patterns of fungal hyphae to anchor the hard‐phase graphene nanosheets within a soft polymer matrix, achieving robust interfacial bonding for the microstructural organization. This approach not only enhances stress transfer and fracture resistance but also introduces self‐monitoring capabilities and lightweight structural properties.

## 2 Results and Discussion

### 2.1 The Fabrication of Layered Bulk Composites (LBCs)

The *Schizophyllum commune* mycelium grows into a 3D fibrous network through the growth pattern being directed layer‐by‐layer (Figures S6 and S7, Supporting Information). After 10‐day growth of *Schizophyllum commune* in the GNs and PVA‐PEG solution (Figure S8, Supporting Information), the mass change of the obtained sample reaches the growth plateau. The increase in sample weight with higher GNs content is primarily due to the fungi's ability to anchor more GNs within the composites. When the *Schizophyllum commune* is grown in PVA‐PEG nutrient solution containing 15 wt.% GNs for five days, black flakes adhere to the surface of the hyphae (Figure 1b). After seven days, the mycelium network becomes denser and the number of black lamellae increases. Finally, after 10 days, a bulk material is generated (Figure 1c). The mycelium‐enabled LBCs also show exceptional self‐regenerability and scalable fabrication ability. When a small portion of the bulk material is placed on a solid culture plate, the mycelium grows autonomously on the surface at room temperature, as shown in Figure S9 (Supporting Information). When the portion is placed into the PVA‐PEG‐GNs solution, it can continue to grow and form new, larger‐sized bulk materials (Figure S10, Supporting Information).

The samples were dried at 50 °C for 12 h and pressed at 5 KPa to form a dense block, and larger‐sized samples, e.g., sizes of 10 cm  ×  10 cm  ×  3 mm, can be prepared (Figure 1d). 3D CT scanning (Figure 1e) shows that GNs are distributed in layers. From the scanning electron microscopy (SEM) images, the top surface also displays such layered structure formed by arranged nanosheets (Figure 1f), which is corroborated by the cross‐sectional images exhibiting the layered structure and the fractured surface showing the stacking of GNs.

### 2.2 Mechanical Properties

**Figure 2:**
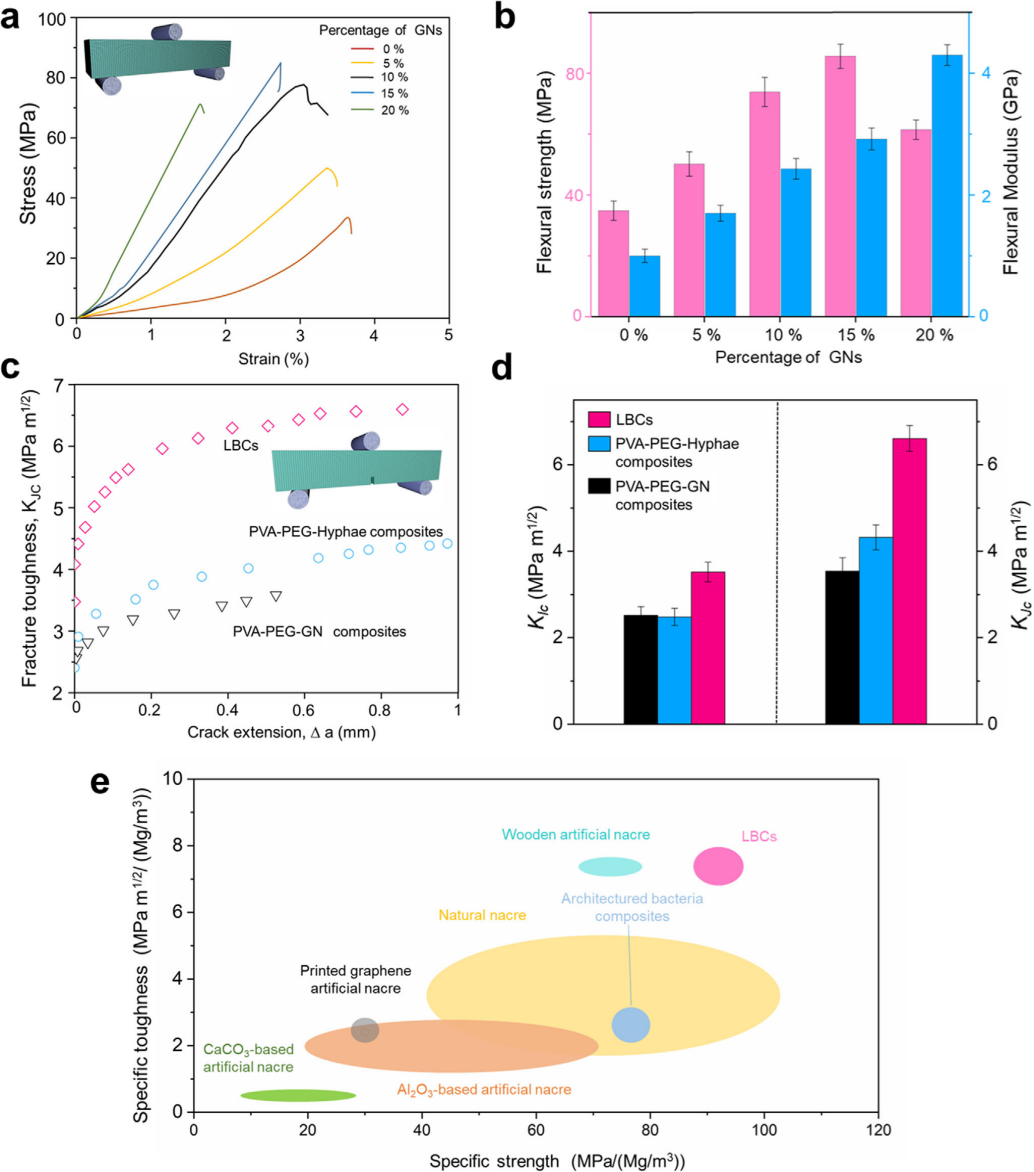
The mechanical properties. Three‐point bending tests of the LBCs with different contents of GNs: a) Typical stress‐strain curves, b) Flexural strength and modulus. c) Crack resistance curve (R curve). The LBCs contain 15 wt.% GNs, and the PVA‐PEG‐GN composites contain 5 wt.% GNs. d) Crack initiation fracture toughness (*K_Ic_*, the left y‐axis) and stable crack propagation (*K_Jc_*, the right y‐axis). The LBCs contain 15 wt.% GNs, and the PVA‐PEG‐GNs composites contain 5 wt.% GNs. Error bars represent the standard deviation of at least five replicate measurements. e) Ashby plot comparing specific strength versus specific toughness for the LBCs (contain 15 wt.% GNs) with representative synthetic engineering materials with ordered layered structure.

Three‐point bending tests to assess the strength and notched three‐point bending tests to characterize the fracture toughness of the LBCs were conducted. The LBCs exhibit different stress‐strain curve behaviors at varying contents of GNs (**Figure** **2**a,b). An optimal strength of 82.6 MPa, a modulus of 3.12 GPa, and a density of 0.89 g cm^−^^3^ are found for LBCs with a content of 15 wt.% GNs. The control samples are prepared with GNs dispersed in the PVA‐PEG matrix with no hyphae growth, which are referred to as PVA‐PEG‐GN composites. The PVA‐PEG‐GN composites exhibit an optimal strength of ≈45 MPa at 5 wt.% GNs (Figure S11, Supporting Information) with a density of 1.21 g cm^−^^3^. Thus, the introduction of hyphae by this interface anchoring strategy can increase the strength of PVA‐PEG‐GN composites. In addition, the composites grown by hyphae in the PVA‐PEG nutrient solution without GNs (called PVA‐PEG‐Hyphae composites) show a strength of 29.5 MPa (Figure 2a,b). This indicates that anchoring GNs at the interface by mycelial hyphae is essential in improving the strength of the composites.

The fracture toughness behaviors and the toughening mechanisms of the LBCs were evaluated by the *J*‐R curve, commonly used for similar synthetic and natural structural materials.^[^ ^33^, ^34^ ^]^ The LBCs exhibit a prominent rising R‐curve behavior (Figure 2c) that is similar to natural layered structural materials (e.g., nacre), indicating their increasing resistance to fracture during crack propagation. From Figure 2d, the LBCs show a *K_Ic_* of ≈3.4 MPa m^1/2^ at the crack initiation and a *K_Jc_* of ≈6.5 MPa m^1/2^ for stable crack propagation, which are both higher than those (*K_Ic_* of 2.4 MPa m^1/2^ and *K_Jc_* of 3.4 MPa m^1/2^, respectively) of the PVA‐PEG‐GN composites (Figure 2c,d). The PVA‐PEG‐Hyphae composites show fracture toughness results of *K_Ic_* of 2.3 MPa m^1/2^ and *K_Jc_* of 4.4 MPa m^1/2^, which are also lower than those of the LBCs. These indicate that leveraging hyphae to anchor the interface between the phases significantly improves toughness in terms of *K_Ic_* and *K_Jc_*.

The specific strength and specific toughness of the LBCs are analyzed, as presented in Figure 2e and Table S4 (Supporting Information). The density of the LBCs is 0.89 g cm^−3^, which is lighter than that of Cristaria Plicata nacre (2.58 g cm^−3^).^[^ ^13^ ^]^ This leads to the specific toughness and specific strength of the LBCs being comparable to those of natural nacre, although the LBCs have a substantially lower content of reinforcement (15% GNs for the LBCs versus ≈95% aragonite for natural nacre). The specific mechanical properties further exceed those of major biomimetic bulk materials with layered structures reported in the literature,^[^ ^13^, ^24^, ^35^, ^36^, ^37^, ^38^ ^]^ including artificial nacre composite materials (wooden, graphene, etc.) and hierarchical structure bacterial cellulose materials. In addition, LBCs have the lowest density compared to most reported layered bulk materials (Table S4, Supporting Information). These results support that the mycelial interface anchoring method can produce lightweight, layered bulk composites with high strength and toughness comparable to natural structural materials. Furthermore, this approach differs from existing popular methods to construct bioinspired layered structural materials,^[^ ^39^, ^40^, ^41^, ^42^, ^43^ ^]^ e.g., ice templating,^[^ ^39^, ^40^ ^]^ 3D printing,^[^ ^38^ ^]^ magnetically assisted,^[^ ^41^, ^42^ ^]^ and self‐assembly.^[^ ^43^ ^]^

In contrast to traditional physical mixing methods, where rigid platelets such as GNs are blended with matrix materials through shear mixing or extrusion, the present work presents a new approach that integrates mycelium growth to fabricate GN‐reinforced composites. This method facilitates the formation of a stable interface between the soft (PVA‐PEG) and the hard (GNs) phases and leverages the self‐organization of the mycelium network to control the positioning and alignment of the GNs. The systematic optimization of parameters such as fungal strain selection, growth medium composition, and environmental conditions ensures uniformity in the distribution of hyphal networks and their interactions with the GNs. As a result, the composites demonstrate enhanced mechanical properties and more reliable performance, distinguishing this approach from conventional physical mixing techniques.

### 2.3 Interface Anchoring

**Figure 3:**
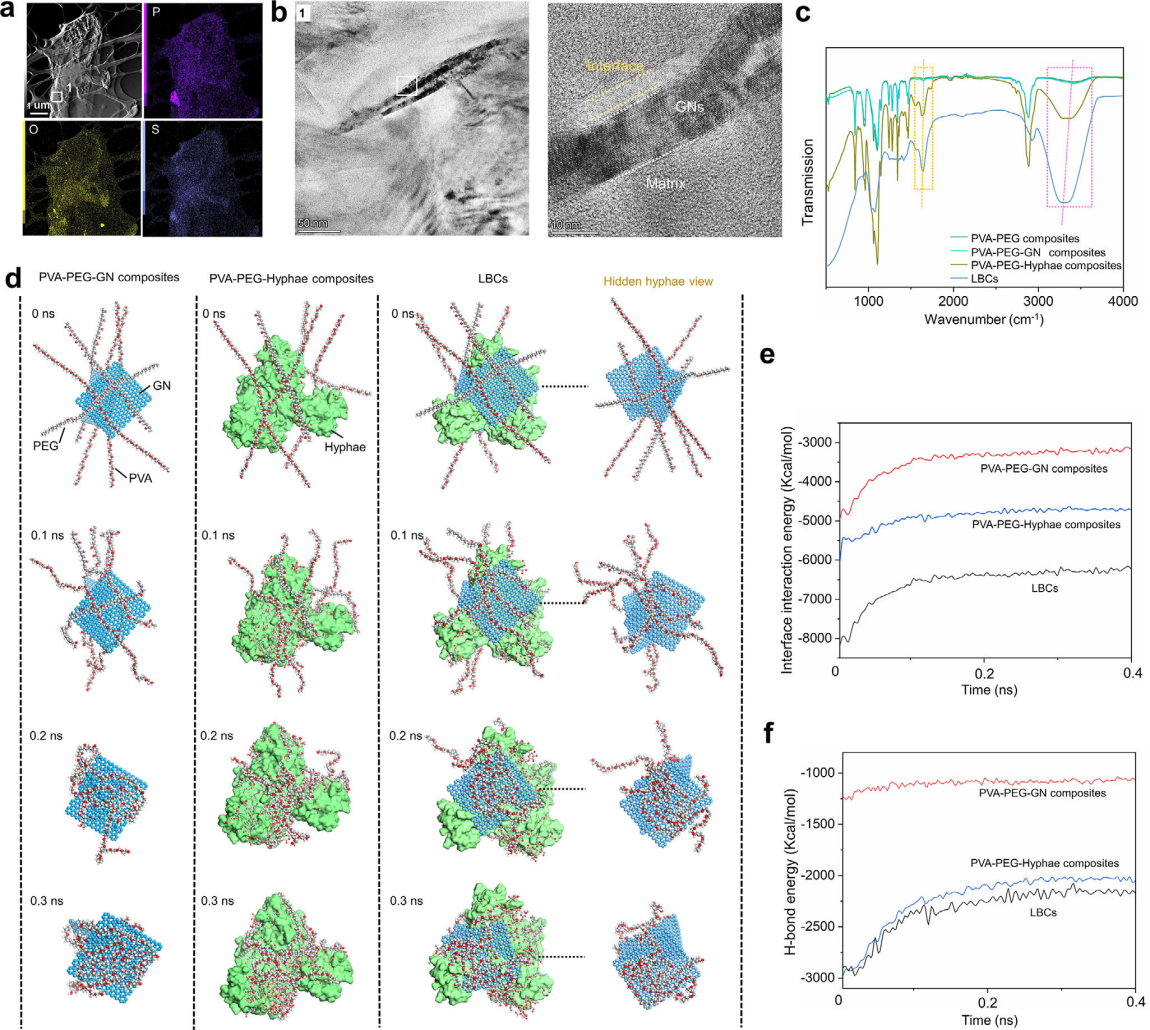
Interface anchoring. a) TEM images and corresponding element distribution of the LBCs. b) The enlarged images of position 1 in (a). c) FTIR analysis. MD simulation results: d) Interface formation behaviors. e) Interface interaction energy. f) Interface H‐bond energy.

The cross‐section of the LBCs was observed by transmission electron microscope, and the results are shown in **Figure** **3**a,b. The main components of S. Commune fungi are polysaccharides, proteins, and phospholipids.^[^ ^28^, ^30^, ^44^ ^]^ From the element distribution of the matrix, there are phosphorus (P), sulfur (S), and oxygen (O) (Figure 3a), which are typical elements of mycelium. We can observe that an interfacial region forms between the GNs and the matrix in Figure 3b. FTIR analysis was performed to investigate the interfacial interactions within the composites (Figure 3c). For the PVA‐PEG and PVA‐PEG‐GN composites, the O─H stretching vibration appears at 3300–3350 cm⁻¹. Upon the introduction of hyphae, the O─H peak in the PVA‐PEG‐Hyphae composites shifts to 3280 cm⁻¹, while in the LBCs, it further shifts to 3250 cm⁻¹. This peek shift indicates the formation of hydrogen bonds between the hydroxyl groups in the PVA‐PEG entanglements and the functional groups in the mycelium (e.g., amine or hydroxyl groups),^[^ ^32^ ^]^ which indicates strengthened hydrogen bonding interactions induced by the hyphae. Additionally, the C═C stretching vibration of the PVA‐PEG‐GN composites is observed at 1580 cm⁻¹ (Figure 3c). After the introduction of hyphae in the LBCs, the C═C peak shifts to 1572 cm⁻¹. The changes in the 1500–1600 cm⁻¹ region, typically attributed to the C═C stretching vibrations of benzene rings,^[^ ^45^ ^]^ may result from π–π interactions between the benzene rings in the hyphae and the GNs.

Molecular dynamics (MD) simulation was used to further reveal the interface formation behavior, and the results are shown in Figure 3d–f. In the PVA‐PEG‐GNs composites, the PVA and PEG chains are entangled with each other on the GN surface. In the LBCs, after the introduction of hyphae, PVA‐PEG chains wrap GNs and approach the hyphae surface, and finally are entangled on the hyphae surface to form an anchored interface. While in the PVA‐PEG‐Hyphae composites, the PVA‐PEG molecular chains approach the hyphae and then are entangled on the surface. During the interface formation process, the absolute value of the interface interaction energy of the LBCs is greater than those of the PVA‐PEG‐GN and PVA‐PEG‐Hyphae composites (Figure 3e). This implies that the LBCs interface is better bonded. It can also be seen that after the introduction of hyphae, hydrogen bonds are formed at the interface. S.Commune fungi contain a large amount of O as well as hydrogen (H) and nitrogen (N), which can effectively form a good interface with PVA‐PEG chains under the action of hydrogen bonds (Figure 3f). There are also a large number of benzene rings in *S. Commune* fungi that can form π‐π interactions with GNs, which further favors the formation of a good interface. A well‐bonded interface can significantly improve the overall strength of the composite material,^[^ ^22^, ^23^, ^24^, ^46^ ^]^ which may be the reason for the high strength of LBCs. The mechanical test results showing a higher strength of the LBCs than those of the PVA‐PEG‐Hyphae and PVA‐PEG‐GN composites prove the effectiveness of the MD simulation.

### 2.4 Toughening Mechanisms

**Figure 4:**
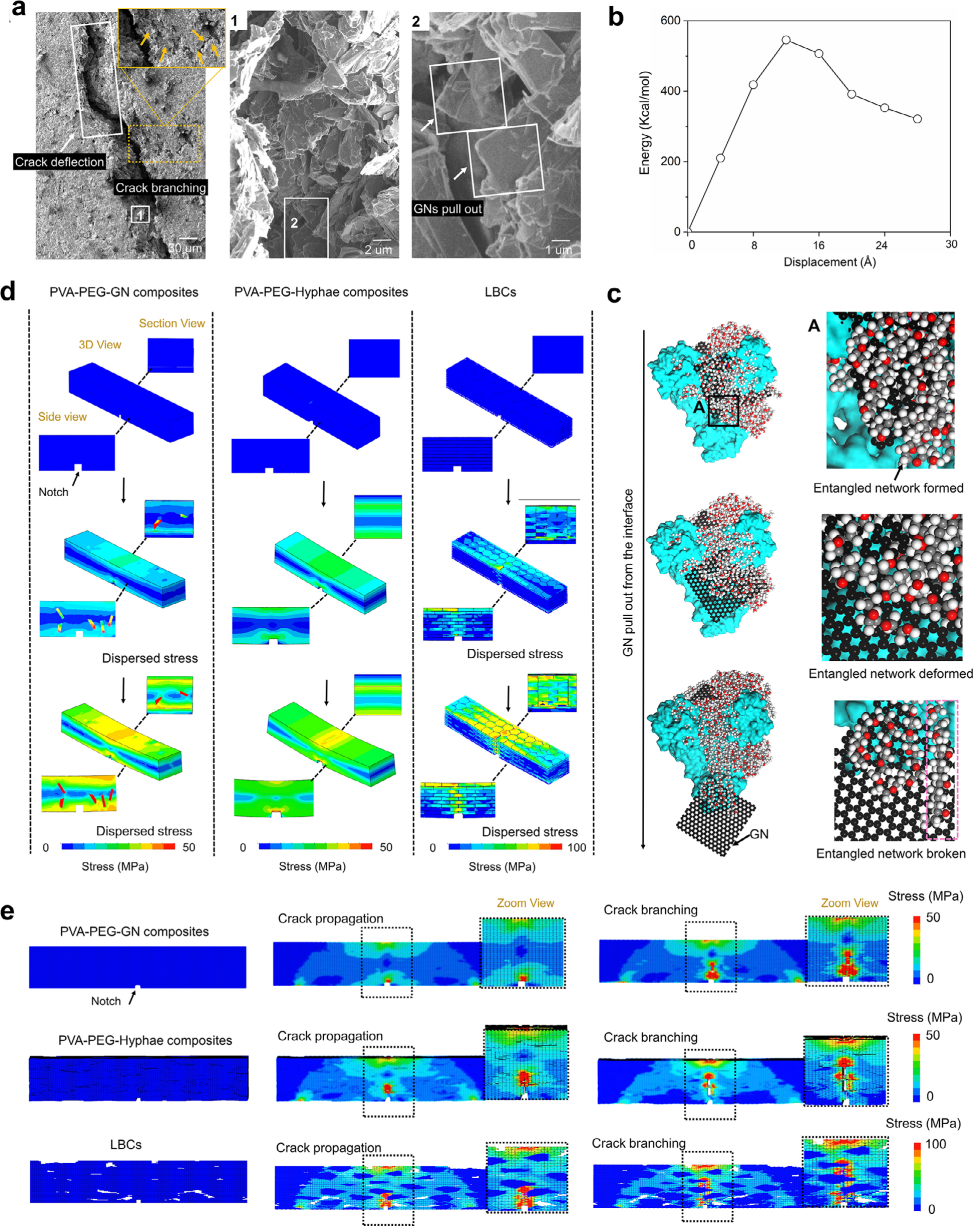
Toughening mechanism. a)The SEM images of the notched three‐point bending fracture morphology. Long‐range crack deflection (white square outline), crack branching (yellow square outline), and GNs pull out (magnified images at 1 and 2). MD simulations: b) Energy changes during GN pull‐out from the interface. c) The GN pull‐out processing and the enlarged image at place A. The GN is pulled out from the interface, accompanied by the deformation of entangled PVA‐PEG molecular chains, and then is straightened and destroyed (pink square). d) Micro‐scale notched flexural simulation. e) Macro‐scale notched flexural simulation.

Fracture mechanics analysis and corresponding theoretical simulations were carried out to investigate reinforcing and toughening mechanisms acting on multiple length scales. The SEM images of the notched three‐point bending fracture morphology are presented in **Figure** **4**a. As observed in the white rectangular area, the crack path exhibited clear deflection at micrometer‐length scales. This is attributed to the layered architecture of the LBCs, which redirects the crack propagation path, thereby increasing energy dissipation. The yellow dashed square shows regions where the crack undergoes branching, which further aids in energy dissipation. In the magnified views of regions 1 and 2 (white square), GNs bridging the crack paths can be observed. These GN bridges act as barriers to crack propagation, enhancing structural integrity by resisting further crack growth and contributing to the fracture toughness of the material. The crack deflection, along with subsequent interface failure, is considered one of the most important extrinsic toughening mechanisms for the high fracture resistance of natural layered materials and other biomimetic structural materials.^[^ ^46^, ^47^, ^48^ ^]^

From the MD simulation of the GN being pulled out from the interface between the PVA‐PEG entanglements and the hyphae (Figure 4b,c), pulling out the GN from the interface deforms the entangled PVA‐PEG molecular chains. Then, these chains are pulled to become straight until the GN is completely pulled out from the interface. This process consumes an amount of energy. The interface separation behavior of the PVA‐PEG and mycelium and the final destruction can be observed from the SEM images of the failure fracture morphologies from the PVA‐PEG‐Hyphae composites (Figure S12a, Supporting Information). The pulling‐out behavior of the GNs from the interface can also be observed from the failure fracture morphologies from the PVA‐PEG‐GN composites (Figure S12b, Supporting Information). The fracture surface reveals an organic layer tightly wrapping the GNs surface, as shown in the enlarged places 1 and 2 in Figure 4a. The magnified SEM images of the fracture surface also support the failure model to be GNs pullout. This verifies the effectiveness of MD simulation in the crack propagation process, which indicates that the frictional sliding of the GNs at the interface and the fracture of the entangled organic matrix contribute to achieving effective energy dissipation. These results suggest that the presence of hyphae at the soft (PVA‐PEG) and hard (GNs) phases enhances the toughness of the LBCs.

The microscale notched three‐point bending finite element (FE) simulation was used to reveal the mechanism of how hyphae at the interface affect material fracture behavior (Figure 4d). When a load is applied to the LBCs, the stress is sustained by the layered GNs and is dispersed layer‐by‐layer along the interface, spreading the stress over a large volume. Compared with PVA‐PEG‐Hyphae composites, the layered GNs can sustain higher stresses and change the crack propagation path for stress de‐localization. For the PVA‐PEG‐GN composites, GNs can sustain loads yet show localized stresses and the stress dispersion is not as effective as the LBCs. This indicates that the layered GNs by mycelium‐enabled interface anchoring sustain higher stresses and allow effective stress dispersion over large volumes via crack deflection, thereby dissipating energy during fracture and improving toughness.

Furthermore, macroscale notched three‐point bending FE simulations were carried out to explore the overall structural failure behavior. From Figure 4e, the cracks in the LBCs start from the notch and propagate along a tortuous path, leading to crack generation and branching behaviors. Compared with the PVA‐PEG‐Hyphae composites and the PVA‐PEG‐GN composites, the layered hard phase plays an essential role in changing the crack propagation path and dispersing stress. These observations further support the notion that the layered structure through the interface anchoring strategy can effectively distribute the load, thereby enhancing the toughness of the bulk material and leading to a rising *R*‐curve behavior.

### 2.5 Impact Resistance

**Figure 5:**
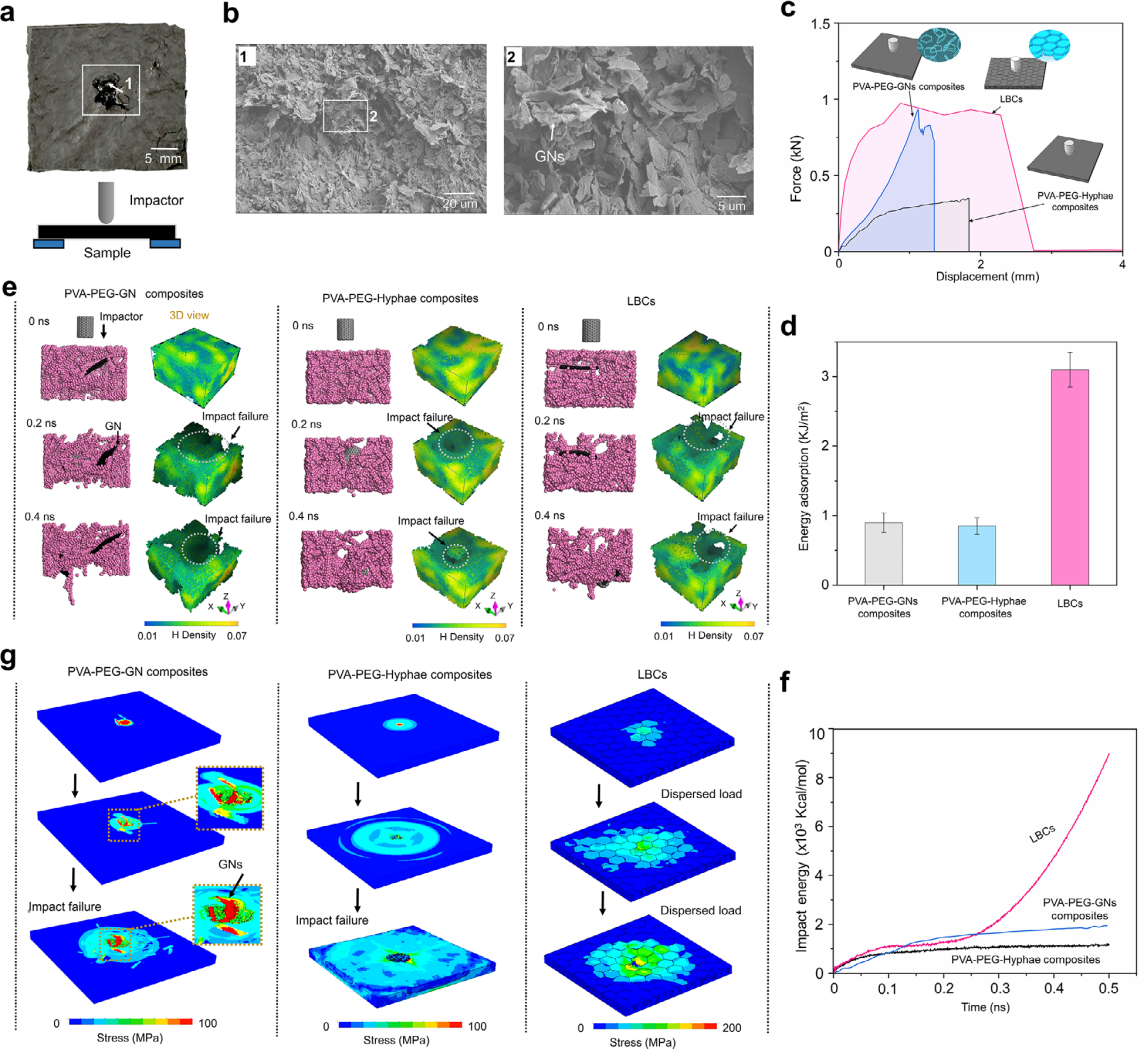
Impact resistance. a) Digital photo of the LBCs sample punctured at an impact velocity of 3 m s^−1^, and the corresponding schematic diagram of the drop tower test system. b) SEM images of the impact fracture at point 1 in (b). c) Force – Displacement curve. d) Total energy, with error bars representing the standard deviation of at least three repeated measurements. Multi‐scale impact simulation results: e) MD impact simulation results and corresponding 3D view H‐atom density distribution. f) The relationship between impact time and energy in MD simulations. g) FE impact simulation results.

The impact resistance of the LBCs was measured using a drop hammer test (**Figure** **5**a), with comparisons to the PVA‐PEG‐GN and the PVA‐PEG‐Hyphae composites. Under an impact velocity of 3 m s^−1^, the LBCs are punctured, yet the overall structure remains intact and no brittle fracture occurs. From the force‐displacement curve shown in Figure 5c, the LBCs exhibits exceptional high strength and larger deformation in buffering impact loads. Compared with the PVA‐PEG‐Hyphae composites, the maximum sustained load significantly increases with also an increase in allowable deformation. The PVA‐PEG‐GN composites show high instantaneous impact force, but the continuous buffering effect is much lower than that of systems with hyphae. This suggests that the load‐bearing behavior and energy dissipation occur more gradually and last over a longer deformation period in the LBCs, indicating an exceptional impact resistance and explaining the remarkable impact energy absorption (Figure 5d) endowed by the hyphae‐induced interface anchoring. From SEM images of the damage position 1 (Figure 5b), the sliding and pull‐out failure behaviors of lamellar GNs from the interface can be observed, demonstrating their important role during impact. The total impact energy of the LBCs is measured at ∼3.1 kJ m^−2^, which is higher than that observed during the brittle failure of natural C. plicata nacre under impact loading, which is ∼1.4 kJ m^−2^.^[^ ^13^ ^]^

The mechanism is investigated by multi‐scale simulations. From MD impact simulation results (Figure 5e), the impact deformation is extensive and the damage depth remains shallow (highlighted by white circles in Figure 5e) in the LBCs and the PVA‐PEG‐GN composites, whereas the PVA‐PEG‐Hyphae composites show limited deformation and damage without sliding and pullout of GN lamellae. More energy is absorbed during impact in the LBCs and the PVA‐PEG‐GN composites. The LBCs exhibit a higher impact energy absorption along a larger deformation under the impact loading (Figure 5c,f). These suggest that the hyphae‐induced interface anchoring effectively blocks the impact load and absorbs energy. FE simulations of the impact failure behavior are presented in Figure 5g. The layered structure of LBCs disperses stress through the interface along the lamellar GNs, absorbing the impact energy efficiently; whereas the PVA‐PEG‐Hyphae composites exhibit single‐path stress transmission and fail. For the PVA‐PEG‐GN composites, the GNs can sustain the load, but there is no effective structure and interface to disperse the load, and failure occurs. The step‐like fluctuations in the force‐displacement curve (Figure 5c) further indicate the occurrence of large‐scale lamellar slip and local microcrack formation within the LBCs. This layered structure by mycelial interface anchoring significantly enhances the energy dissipation capabilities of the LBCs.

### 2.6 Applications in Health Monitoring Structural Materials

**Figure 6:**
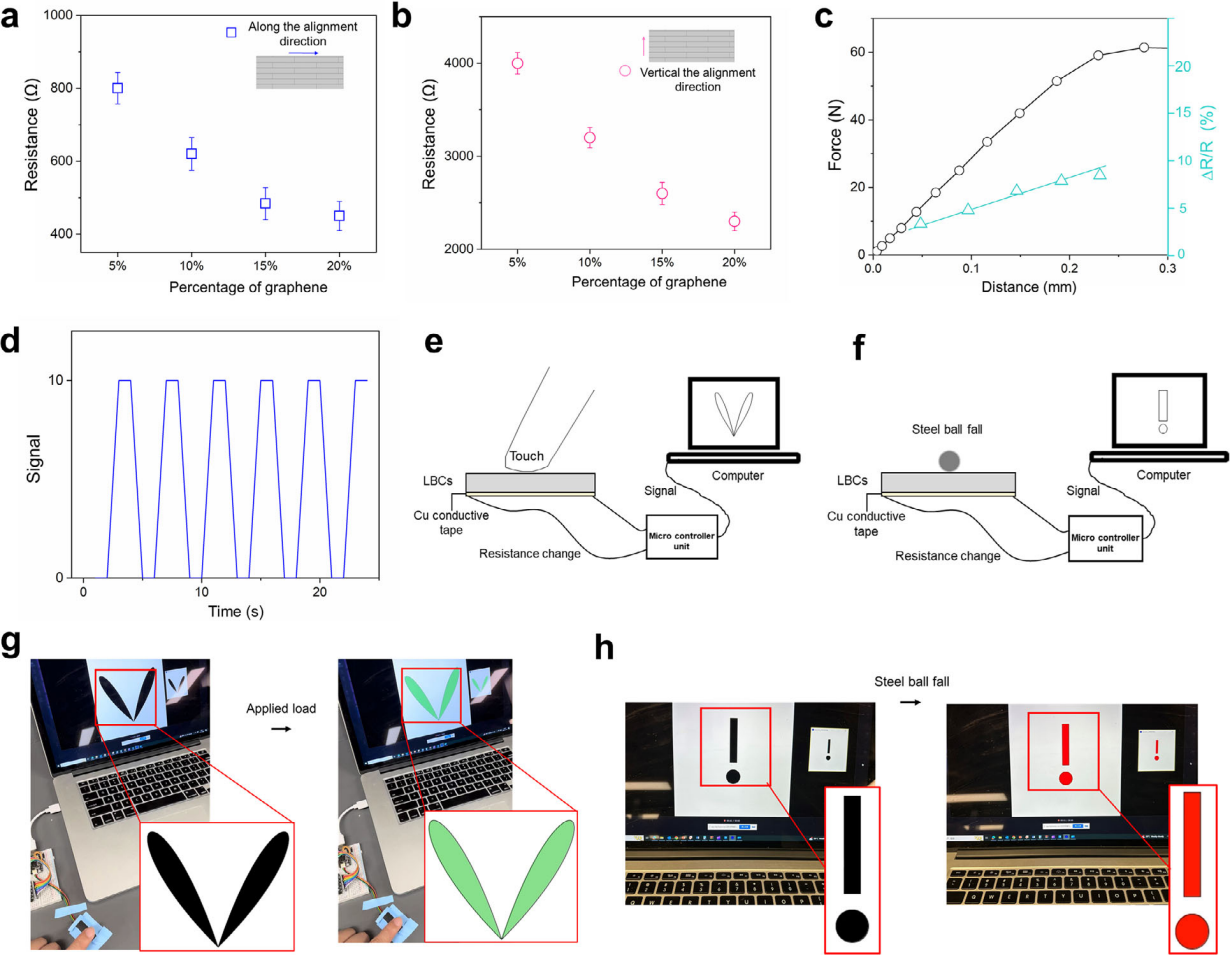
Applications in health monitoring structural materials. Changes in resistance of the LBCs with different contents of GNs: a) Along the alignment direction, b) Vertical to the alignment direction. c) Changes in resistance with external load. d) Relationship between finger action time and electrical signal response change. e,f): Health monitoring conceptual designs. e) Monitoring quasi‐static forces, the color of the pattern on the computer screen changes when the material is pressure‐loaded. f) Monitoring impact forces, the impact by a falling object induces changes in the color of the pattern on the computer screen. The experiment results: g) When the load is applied to the LBCs, the heart changes from black to green, h) When the LBCs are struck by the steel ball, the exclamation mark changes from black to red.

When the distribution of graphene in the material is layered, conductivity improves.^[^ ^18^, ^19^, ^20^, ^21^ ^]^ To evaluate the potential application of the LBCs (15 wt.% of GNs) in health monitoring structural materials, the electrical conductivity was measured. The LBCs exhibit a conductivity of ≈200 S m^−1^ in the longitudinal direction, as shown in Table S5 (Supporting Information), which is higher than the conductivity of the PVA‐PEG‐GN composites prepared by the common blending method (0.0001 S m^−1^) (Table S6, Supporting Information)). The piezoresistive response of the LBCs is characterized, as shown in **Figure** **6**a–c. The resistance of the LBCs in the direction parallel to the GNs alignment is generally lower than that of the LBCs in the direction perpendicular to the GNs alignment (Figure 6a,b). This anisotropic electrical behavior is attributed to the fast electron transport parallel to the alignment direction of adjacent GNs, but electron transport is blocked by the insulating organic matrix in the direction perpendicular to the aligned GNs.

An RC circuit was built and a digital microcontroller was used to measure the resistance change of the LBCs during external loadings. The relationship between the resistance change and the external load change is established, as shown in Figure 6c. When the pressure increases, the LBCs resistance change increases. The GNs having excellent electrical conductivity are arranged in alternating layers in the LBCs. The GNs respond to mechanical strain by altering their alignment and thus connectivity within the composites, thereby modulating the resistance. The growth pattern of fungi forms a flexible, self‐organizing network. When subjected to deformation, the network composed of PVA, PEG, and Hyphae preserves its structural integrity, transmitting mechanical stress and regulating charge transfer, which further amplifies the piezoresistive effect. We transfer this change in resistance to the computer, and convert the signal changes into a graphic display interface. Upon applying loads, the LBCs respond quickly, as depicted in Figure 6d and Video S1, (Supporting Information). We demonstrate the health monitoring function of the LBCs through finger‐applied pressure loadings (Figure 6e) and impact forces from free‐fall objects (Figure 6f), then convert the signal change into a visual color change on the screen. The black heart image on the computer screen turns green (Figure 6g; Video S2, Supporting Information), showing the response when the finger presses the LBCs. The exclamation mark image on the computer screen changes from black to red (Figure 6h; Video S3, Supporting Information), indicating being hit by a falling steel ball. Therefore, the integration of mechanical properties, such as high toughness, specific strength, impact resistance, and electrical conductivity, makes the LBCs particularly suitable for structural health monitoring applications.

## 3 Conclusion

In this study, we proposed an interface anchoring strategy, fixing the interface between the soft and hard phases to immobilize 2D materials, by leveraging the growth of biological living cells. This approach resulted in the formation of a lightweight and strong composite material (the LBCs) with a layered structure composed of soft and hard phases. The mechanical properties of the LBCs are comparable to those of the hierarchical layered materials, including natural nacre and artificial biomimetic composite materials. The effectiveness of the interface design strategy in improving strength and toughness is revealed through multi‐scale simulations. Furthermore, it exhibits good electrical conductivity and health monitoring functions under external force stimulations, suggesting its potential application as anti‐collision materials in sports and aerospace industries. Our strategy and manufacturing method are not only environmentally friendly and scalable but also differ from most existing techniques (such as the ice‐template method, shear force, magnetic field, and electric field) used for assembling 2D materials into 3D structures, which often require external forces or templates to organize components. This approach offers several advantages, including scalability, environmental sustainability, and the ability to leverage living biological elements, thereby contributing to lightweight, self‐regenerative properties, enhanced mechanical properties, and self‐sensing functional capabilities. Additionally, challenges remain in resolving biological growth timing and achieving precisely controllable periodic structures which require further studies.

## 4 Experimental Section

The methods are available in the Supporting Information.

## Conflict of Interest

The authors declare no conflict of interest.

## Author Contributions

H.W. performed conceptualization. H.W., J.R.L., Z.Y.W., X.F.C., K.J., J.T., and, B.W. performed methodology and wrote reviewed, and edited. H.W., J.R.L., and, Z.Y.W. performed software. H.W. and J.T. performed an investigation. J.T. and B.W. performed supervision and resources. H.W. wrote the original draft.

## Supporting information



Supporting Information

Supplemental Video 1

Supplemental Video 2

Supplemental Video 3

## Data Availability

Data sharing is not applicable to this article as no new data were created or analyzed in this study.
