# Supplementary material for: Bioinspired Strong and Tough Layered Bulk Composites via Mycelial Interface Anchoring Strategy
Source: Adv Sci (Weinh). 2025 Mar 24;12(19):2413226. doi: 10.1002/advs.202413226 (PMC12097066; doi:10.1002/advs.202413226)
Supplement: Supplementary file 1 — Supporting Information [file ADVS-12-2413226-s003.docx]

**Supporting information**

**Bioinspired strong and tough layered bulk composites via mycelial interface anchoring strategy**

Hao Wang^1^, Jurui Liu^1^, Zhangyu Wu^2^, Xianfeng Chen^3^, Kai Jin^4*^, Jie Tao^5*^, Bin Wang^1*^

^1^Department of Mechanical Engineering, City University of Hong Kong, Hong Kong

^2^School of Materials Science and Engineering, Southeast University, Nanjing, China

^3^A*STAR Quantum Innovation Centre (Q.InC), Institute for Materials Research and Engineering(IMRE), Agency for Science,Technology and Research(A*STAR), Singapore

^4^School of Materials Science and Engineering, Ocean University of China, Qingdao, China

^5^College of Materials Science and Technology, Nanjing University of Aeronautics and Astronautics, Nanjing, China

*Corresponding Author: jinkai@ouc.edu.cn, taojie@nuaa.edu.cn, bin.wang@cityu.edu.hk

**This file includes:**

Materials and Methods (text, Figures S1 to S5, and Tables S1-S2)

Supportive results (text, Figures S6-S14, and Tables S4-S6)

Code for visualizing resistance change in computer

Captions for Videos S1 to S3

**Other Supplementary Information for this manuscript include the following:**

Videos S1 to S3

**Methods**

**Materials**

The following chemicals were purchased from Sigma-Aldrich and used: malt extract, peptone, agar, D-glucose, yeast extract, NaC_5_H_8_NO_4_, KH_2_PO_4_, K_2_HPO_4_, MgSO_4_, polyvinyl alcohol (PVA, M_w_ 89000-98000, 99+% hydrolyzed), and Polyethene glycol (PEG, average mol wt 100000, powder). The graphene nanosheets (GNs) were procured from the American Graphene Supermarket, with an average flake thickness of approximately 8 nm (equivalent to 20–30 graphene monolayers) and an average lateral particle size of ~5 μm. The fungi of *Schizophyllum commune* were cultured for experimental use.

**Culture solution for hyphae growth**

Solid media: Prepare malt extract agar by dissolving 15 g agar, 10 g peptone, and 30 g malt extract in 1 L deionized water. Graphene-PVA-PEG liquid culture medium: Dissolve the following ingredients in 250ml water: D-glucose: 3g, KH_2_PO_4_: 0.5g, K_2_HPO_4_: 0.04g, MgSO_4_: 0.1g, PVA: 2g, PEG: 2g. Adjust the ratio of graphene flakes to the PVA-PEG mixture ranging from 0% to 20% in 5% increments. Prior to autoclaving, these mixtures were homogenized by ultrasonic stirring to disperse the additives.

**The preparation of layered bulk composites (LBCs)**

Use the procedure described above to formulate PVA-PEG liquid nutrient solutions containing graphene with additive weight ratios ranging from 0% to 20%. *Schizophyllum commune* were then introduced into a 1 L flask containing 250 ml of liquid culture medium. Place the flask in a controlled environment with a relative humidity of 50% and a temperature of 23°C. Continuous shaking at 120 rpm was initiated and maintained under these conditions for 7 to 15 days, and growing bulk materials were collected. For the dry samples, treat at 50 °C for 24 hours to remove the moisture, and perform 5kN pressure densification for 24 hours. All samples were pressed in a mold with dimensions of 10cm × 10cm ×2.5mm.. For the PVA-PEG-Hyphae composites, the preparation method is the same as the LBCs, but there are no GNs in the nutrient solution. For the composites without hyphae, referred to PVA-PEG-GNs, 25 ml of GNs solution with different concentrations is prepared, the mass fraction of GNs is 0%-20%, and the interval is 5%. The weight of PVA is 2g, and PEG is 2g. First, the GNs-PVA solution is ultrasonically treated for 30 minutes, stirred at 100 °C for 1 hour, and then PEG is added and stirred at 100°C for 1 hour, and dried in a vacuum oven at 50°C for 12 hours. The following important parameters are outlined to control the growth of the mycelium-based composite:

Fungal strain selection: Choosing the appropriate fungal species is crucial to achieving the desired network morphology. For example, *Schizophyllum* was selected for their ability to produce distinct types of hyphal networks (compact or branched) that are suited for different applications.

Growth medium composition: The carbon and nitrogen sources in the growth medium influence the density and morphology of the fungal network. The medium for each specific application is optimized to achieve controlled growth and integration with the polymer matrix.

Temperature and humidity control: Maintaining an optimal temperature (e.g., 20-30°C) and humidity levels (e.g., 70-90% RH) is essential for promoting consistent fungal growth and ensuring uniform network formation.

Growth time: The growth time is adjusted based on the required network density. A balance is achieved between growth rate and network control to maintain material stability.

Graphene nanosheets (GNs) distribution: The concentration and uniform dispersion of GNPs within the matrix are key to ensuring effective interaction with the growing mycelium network. Specific GNP dispersion techniques such as sonication are employed to ensure even distribution.

Drying and compression: After fungal growth, drying at controlled temperatures (50-70°C) and applying pressure (5-10 KPa) helps consolidate the material and fix the network in place.

Post-growth stabilization: A final curing step (e.g., 24-48 hours at 70°C) ensures the stability and longevity of the material.

**Morphology and composition analysis**

The samples were visualized by optical imaging using a Zeiss Axio Scope A1 microscope manufactured by Zeiss. A gold coating with a thickness of 5 nm was applied to the sample prior to imaging. A ZEISS Xradia 610 Versa 3D X-ray microscope was used for 3D scan samples. The morphology of the samples were characterized using a Helios 5 dual-beam scanning electron microscope (FIB-SEM). Transmission electron microscopy (TEM) analysis was performed using a JEOL 2100F instrument. FTIR absorption spectra were recorded using a Shimadzu IR Tracer 100 spectrometer with a wavenumber range from 4000 to 400 cm^−1^ and a resolution setting of 2 cm^−1^, accumulating data from 64 scans to improve accuracy. Wide-angle X-ray scattering (WAXS) was performed on a XEUSS WAXS/SAXS system (Xenocs, France) equipped with a rotating anode X-ray source. All tested samples were characterized along the cross section of the sample. To calculate the density of the produced material, the sample was first heated to 50°C to evaporate any residual water. The sample’s weight was measured using an analytical balance. The volume of the sample was determined using its geometrical dimensions, as the samples were cut into regular shapes. Density (g/cm^3^) was calculated by dividing the weight by its volume.

**Mechanical test**

In the three-point bending test (conducted on at least three samples), a support span of 10 mm was used, with a loading point size (hemispheric radius) of approximately 3.2 mm, and a bending displacement rate of 0.5 mm/min. For single-edge notch bending (SENB) testing, specimens with dimensions of d ≈ 1 mm and b ≈ 2 mm were notched to approximately 30% of their width using a 150 µm thick diamond blade. The notches were then refined by repeatedly swiping with a knife blade to achieve a final notch radius of approximately 30 µm. SENB testing, which involved a minimum of three samples, was conducted at a consistent displacement rate of 0.05 mm/min. Both the three-point flexural strength and SENB tests were performed using a universal testing machine (Instron 5689, Instron Corp., USA), following the guidelines provided by ASTM D790-03. At least 5 samples were tested in each group.

The stress (𝜎) and strain (𝜀) in a three-point bending test were calculated using the following equations:

$\sigma=\frac{3FS}{2bd^{2}}$ (1)

$\varepsilon=\frac{6Dd}{S^{2}}$ (2)

where $F$ is the force at the point of failure, $D$ is the displacement at the point of failure, $b$ and $d$represent the specimen’s width and thickness, $S$ is the length of the support span.

The fracture toughness, *K_IC_*, under plane strain conditions was calculated using the following equations [1]：

$K_{IC}=\frac{P_{IC}f\left( a/W \right)}{BW^{3/2}}$, $x=a/W$ (3)

$f\left( x \right)=\frac{3{a/W}^{1/2}\left[ 1.99-x\left( 1-a/W \right)\left( 2.15-3.93a/W+2.7{(\frac{a}{w})}^{2} \right) \right]}{2\left( 1+2a/W \right)\left( 1-a/W \right)^{3/2}}$ (4)

where $P_{IC}$ is the maximum load, $B$ and $W$ are the width and height of the specimen, and $a$ is the initial crack length.

The fracture toughness, *K_Jc_*, was evaluated using J-integral calculation, which considers both elastic and plastic contributions. This method aligns with established approaches used to estimate the properties of various bioinspired composites.

$J=J_{el}+J_{pl}$ (5)

where $J_{el}$ is the elastic contribution on the basis of linear elastic fracture mechanics,

$J_{el}=\frac{K_{IC}^{2}}{E^{'}}$ (6)

where the plastic contribution, $J_{pl}$ can be calculated with the following equation:

$J_{pl}=\frac{{2A}_{pl}}{B(W-a)}$ (7)

where $A_{pl}$ is the plastic area underneath the load–displacement curve, $J$ values can be transformed into $K$ values by the following equation:

$K_{Jc}=({JE')}^{1/2}$ (8)

in which $E' = E(1 - v^{2})$, where $E$ represents Young's modulus and $v$ denotes the Poisson ratio. It should be noted that the influence of the variation in $E$ on $K_{JC}$ is relatively limited. Therefore, in this context, $E'$can be effectively replaced by $E$.

The SENB method utilizes the equivalence between compliance and crack length to analyze toughness. This achieved is by computing compliance using the formula $C=\frac{u}{f^{,}}$, where $u$ represents crack propagation, and $f^{,}$ denotes the forces at each point after the crack has propagated beyond that point. Subsequently, a recursive process is applied to calculate the crack length, as follows:

$a_{n}=a_{n-1}+\frac{W-a_{n-1}}{2}\times\frac{c_{n}-c_{n-1}}{c_{n}}$ (9)

$c_{n}=\frac{u_{n}}{f_{n}}$ (10)

$\Delta a=a_{n}-a$ (11)

where $W$ is the width of the sample, $a$ and $c$ are the crack length and compliance, respectively calculated at the $n$ and $n-1$ steps, and $\Delta a$ is the amount by which the crack extends.

**Impact test**

A drop tower impact system (MTS Industrial Systems) was employed to investigate the impact properties of the samples. The sample was simply supported on a 5 mm wide shoulder machined on the frame. A hemispherical impactor with a diameter of 3 mm and a mass of 0.5 kg was released from a height to achieve an impact velocity of 3 m/s. The energy adsorption (E) was calculated as the area enclosed within the loop of the force-displacement curve.

**Protective warning functions test**

The conductivity was measured using the four-probe method, and the instrument was the RS Pro Digital Multimeter. The micro-controller (Arduino) was used to realize electrical signal recognition and visualize the signal changes in laptop by using Processing. The diameter of the steel ball is 30 mm.

**Multiscale simulation**

**Molecular dynamics (MD) simulation**

Molecular dynamics (MD) simulation is an effective tool for describing intermolecular behavior and interaction mechanisms at the molecular scale. It has been widely used to elucidate the physical mechanisms of biological materials [2, 3]. We utilize MD simulations to investigate the interface formation mechanism.

**Modeling**. A model of the graphene-PVA-PEG and hyphal interface formation process was developed. Initially, a mycelium model was constructed based on previous studies [4], which showed that mycelium is mainly composed of hyphae. The hyphae model is shown in **Fig. S1**. Chitin is the main component of polysaccharide (about 60%), and Mannan and β-glucan are the main components of protein (about 30%), and phospholipids are the main components of the hyphal cell membrane (about 10%). The hyphal model incorporates all relevant functional groups to accurately represent intermolecular reactions. Subsequently, a hyphae simulation model considering structural organization and mass fraction was established, and the box size was enlarged to 15 nm x 15 nm x 15 nm. To create a hybrid model, a Monte Carlo method was used to introduce PVA-PEG molecules and graphene around the hyphal model. Structural optimization and dynamic calculations were then performed to obtain the final stable structure, representing a hybrid model of hyphae with PV-PEG-graphene.

**Calculating**. Initial simulations were performed using the Molecular Dynamics (MD) and Polymer Consistent Force Field (PCFF) atom-molecule force fields in LAMMPS [5]. The simulation protocol involves 5000 conjugate gradient energy minimization steps followed by 100 ps simulations at constant temperature (300 K) and constant volume. Subsequently, 100 ps equilibration was performed at constant temperature (300 K) and constant pressure (1 bar).

To describe intermolecular interactions, the LJ 12_10 potential was used to calculate van der Waals forces and hydrogen bond interactions. The energy calculations were carried out using the following formulas (12) and (13):

$E_{vdw}=D_{0}\left[ 5{\frac{R_{0}}{R}}^{12}-6{\frac{R_{0}}{R}}^{10} \right]$ (12)

where $D_{0}$ is equilibrium well depth (Kcal/mol), $R_{0}$ is equilibrium distance (Å), $R$ is the initial distance (Å).

$E_{h-bond}=D_{0}\left[ 5{\frac{R_{0}}{R}}^{12}-6{\frac{R_{0}}{R}}^{10} \right]\left( \cos\emptyset\right)^{4}$ (13)

where $D_{0}$ is equilibrium well depth (Kcal/mol), $R_{0}$ is equilibrium distance (Å), $R$ is the initial distance (Å) , the torsion angle $\emptyset$ formed by four particles, measured in radians.

For the calculation of graphene pulling out from the interface, a constant force was applied to the graphene and the graphene is pulled out along the Z direction with a time of 0.2 ns and a speed of 0.1 Å/ps.

For the impact simulation, a cylindrical rigid body with a size of 0.1 nm was first created and impacted the LBCs (size of 1 nm x 1 nm x 6 nm) as a speed of 0.3 Å/ps.


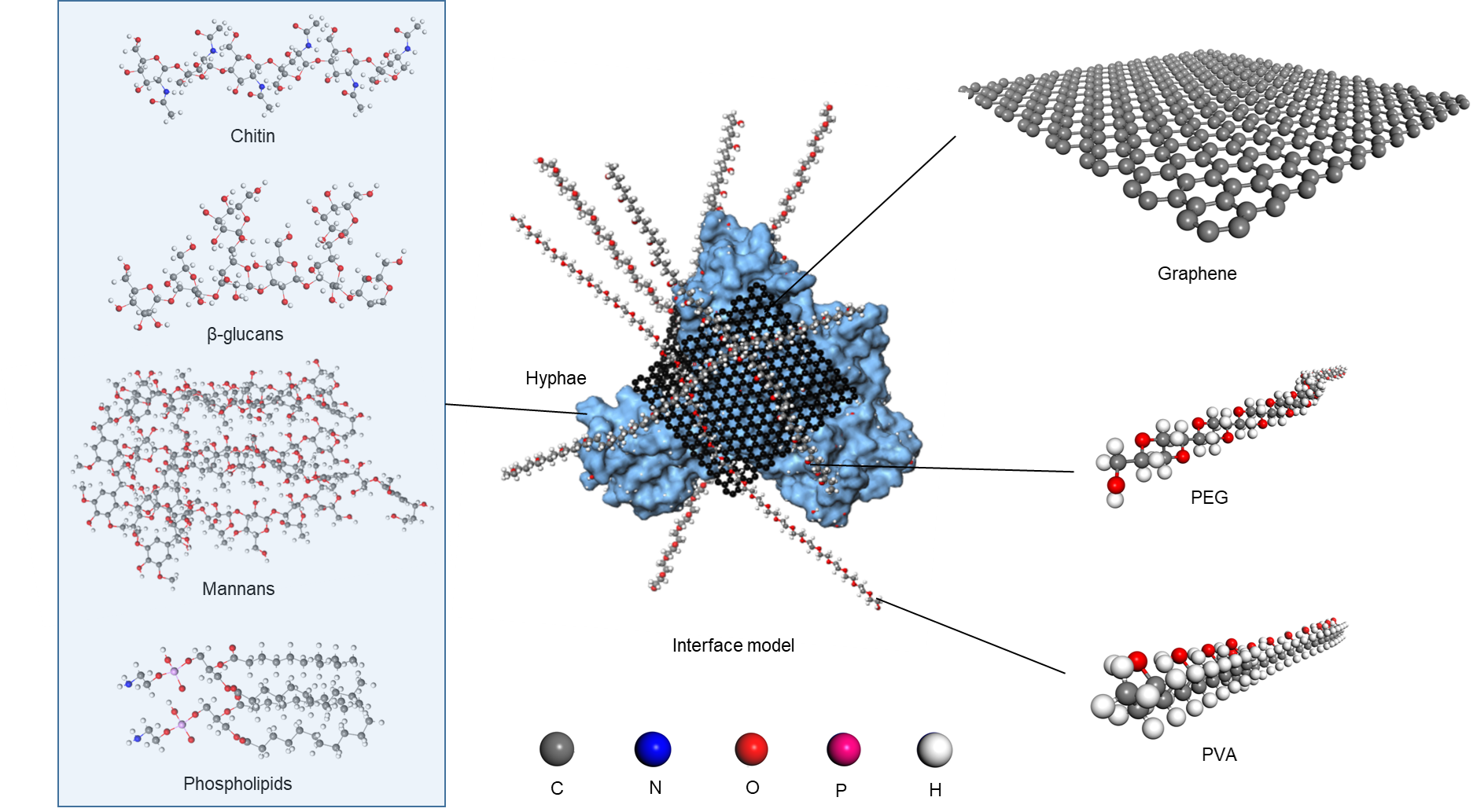


**Fig. S1** **MD simulation model.** The initial state of the interface between hyphae and PVA-PEG-graphene. The hyphae is composed of chitin, β-glucan, mannan, and phospholipids. Graphene nanosheet (GN) containing six-membered benzene rings.

**Finite Element (FE) simulation**

**Microscale Modeling.** To create the geometry of the layered graphene nanosheets (GNs) model, a python script based on Voronoi diagrams was adapt [5]. When nodes were evenly distributed at the vertices of equilateral triangles, the resulting Voronoi diagram will consist of uniform equilateral hexagons, as shown in **Fig S2a**. The single layer of the GNs is extruded the 2D Voronoi of plane direction to obtain with specified thickness 0.015 mm. There is total 10 layers GNs throughout the thickness of the specimen. The center of adjacent layers is located at the intersection of adjacent layers, which ensures the staggered distribution of the GNs structure, as illustrated in **Fig. S2b**. These processes were all implemented through python script. The zero-thickness cohesive elements were inserted into the junction and interlayer of the GNs to simulate the resin.

The PVA-PEG-GNs models for inclusions were developed as a comparison with a regular distribution model to analyze the effect of structure on stress and displacement distributions. The generation of the inclusions and the GNs random distribution is achieved by Python script. The random distribution models are shown in the **Fig S3a**.


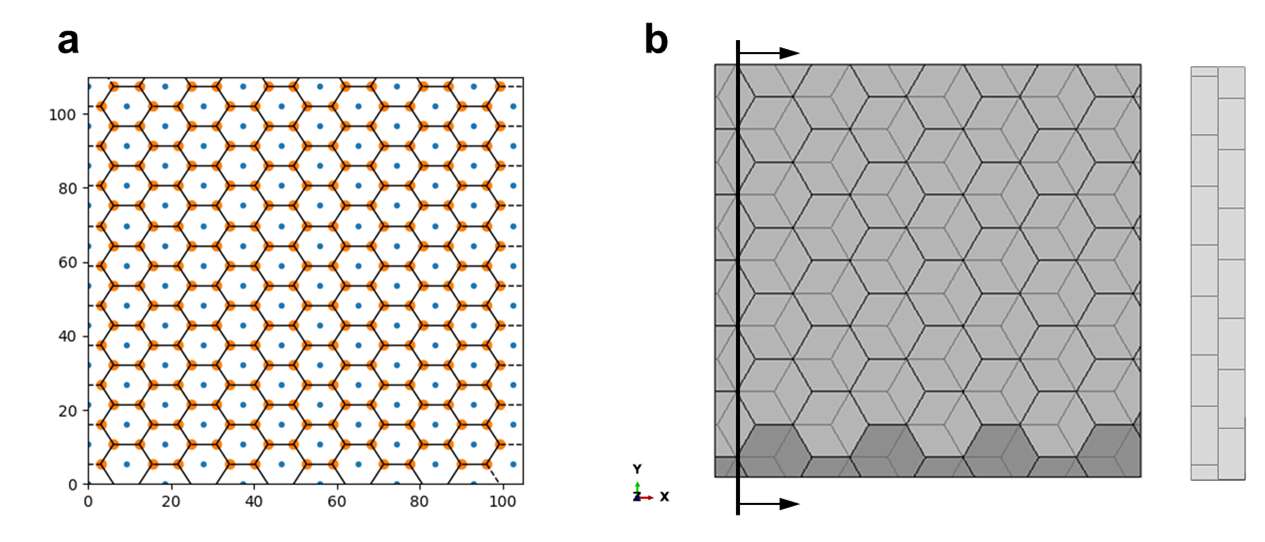


**Fig S2** **Layered graphene nanosheets (GNs) model.** **a**. Voronoi diagram in a grid formation. **b**. The schematic of graphene lay-up method.

To further investigate the deformation and fracture mechanism of the LBCs, 3D two-phase mesoscale models with dimensions of 25 mm × 2 mm × 1.5 mm was developed by using the software ABAQUS v2022. These model feature a staggered arrangement of stiff graphene platelets bonded by a soft organic interlayer, as depicted in **Fig. S3**. For three point bending test simulation, according to the real experimental program, a static load was applied to a rigid rod that was mounted on the mid-span of the specimen. Two rigid supports were positioned at the specimen's base, with a 10 mm distance between them. For the impact test simulation, the bottom surface of the samples is fixed and displacement boundary condition was applied to the drill with the speed of 3m/s.


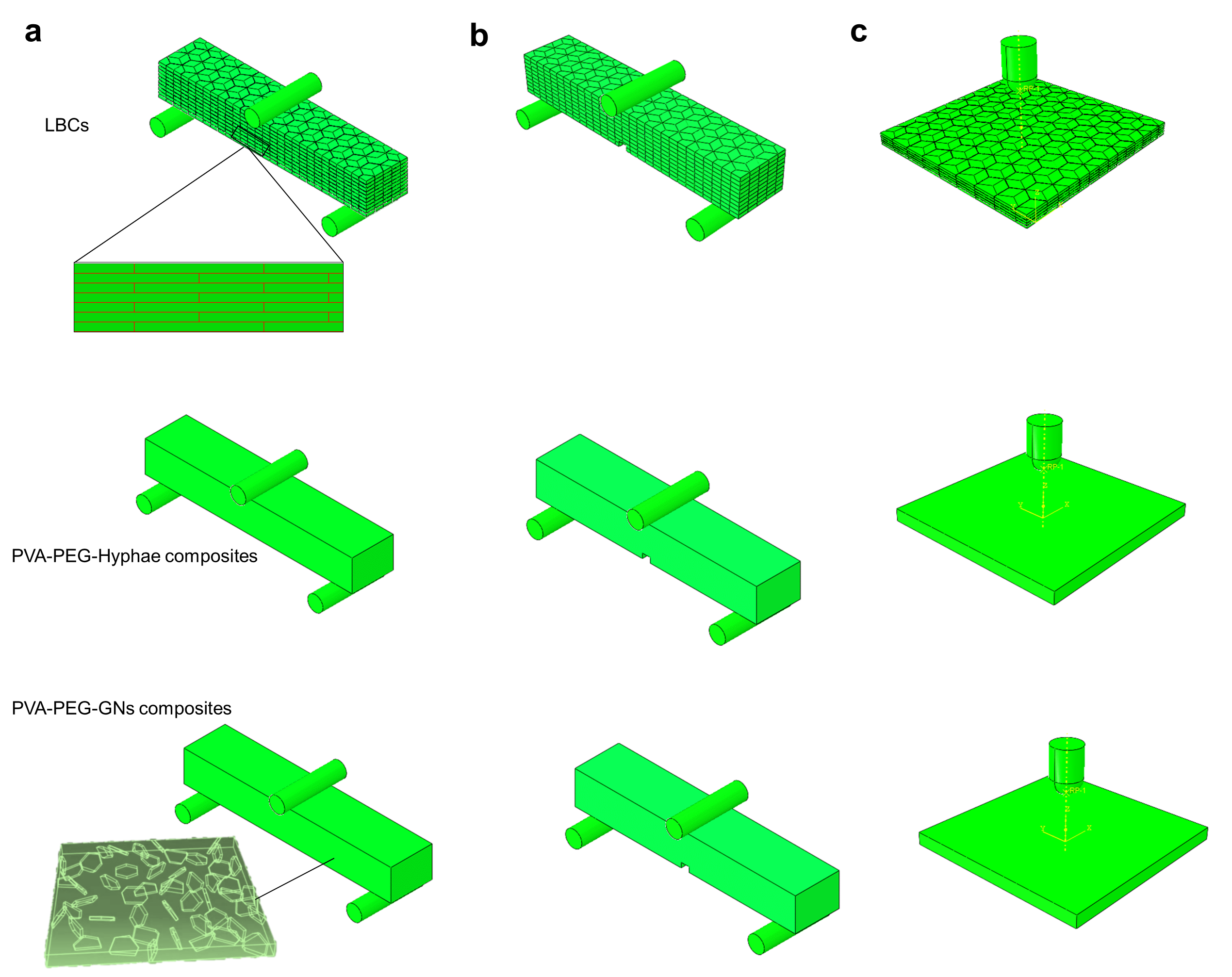


**Fig. S3** **Microscale FE model.** **a**. Three-point bending. b. Notched specimen under three-point bending. **c**. Impact.

**Calculating.** The basic material parameters of s graphene sheets are listed as follows: Young’s modulus E = 1.02 TPa and Poison ratio v=0.1, tensile strength ft = 120 GPa. For simulating the brittle failure manner of the graphene sheets during the loading process. The maximum stress failure property was set to graphene. The parameters of material properties are list in **Table S1**.

**Table S1** The material properties of the graphene phase.

| Properties | Value | Units |
| --- | --- | --- |
| Density | 2.267 | g/cm^3^ |
| Young’s modulus | 1.02 | TPa |
| Position’s ratio | 0.16 |  |
| Strength | 120 | GPa |

**Table S2** The material properties of the organic phase.

| Properties | Value | Units |
| --- | --- | --- |
| Density | 0.85 | g/cm^3^ |
| Young’s modulus | 3510 | MPa |
| Position’s ratio | 0.36 |  |
| Strength | 48 | MPa |

The failure behavior of the organic phase was simulated by zero-thickness cohesive elements between the ordered arrangement graphene sheets. The stress-strain response of cohesive elements is defined by Equation (14).

$\left\{\begin{matrix} \tau_{n} \\ \tau_{s} \\ \tau_{t} \end{matrix}\right\}=\left[\begin{matrix} K_{n} & 0 & 0 \\ 0 & K_{s} & 0 \\ 0 & 0 & K_{t} \end{matrix}\right]\left\{\begin{matrix} \delta_{n}/T_{n} \\ \delta_{s}/T_{s} \\ \delta_{t}/T_{t} \end{matrix}\right\}$ (14)

where 𝜏_𝑛_ , 𝜏_𝑠_ , and 𝜏_𝑡_ represent stresses in three spatial directions, while 𝐾_𝑛_ , 𝐾_𝑠_ , and 𝐾_𝑡_ denote stiffness in different orientations. The maximum displacement values are represented by 𝛿_𝑛_, 𝛿_𝑠_, and 𝛿_𝑡_, and 𝑇_n_, and 𝑇_𝑡_ signify the calculated thicknesses of the cohesive elements.

When the node separation distance exceeds $\delta_{e}^{i}$, the cohesive elements enter the damage evolution stage, accompanied by a reduction in the stiffness of cohesive elements. The stress-strain relationship for the post-damaged elements is illustrated in Equation (15).

$\tau_{i}=\left(1-D_{i}\right)K_{i}\delta_{i}/T_{i}=K_{i}^{'}\delta_{i}/T_{i} i=n,s\text{ and }t$ (15)

The parameter D denotes the damage factor of the cohesive element, with D=0 and D=1 corresponding to the intact and complete failure of the element, respectively. The determination of the damage factor for the cohesive element in the case of a bilinear ontological relation is outlined in Equation (16).

$D_{i}=\left\{\begin{matrix} 0 & 0\leq\delta_{i}<\delta_{e}^{i} \\ \frac{\delta_{f}^{i}\left(\delta_{i}^{\max}-\delta_{e}^{i}\right)}{\delta_{i}^{\max}\left(\delta_{f}^{i}-\delta_{e}^{i}\right)} & \delta_{e}^{i}\leq\delta_{i}<\delta_{f}^{i},i=n,s\text{ and }t \\ 1 & \delta_{i}>\delta_{f}^{i} \end{matrix}\right.$ (16)

The maximum stress criterion is assumed to cohesive element to evaluate initial damage, as represented in Equation (17).

$f=\max\left\{\frac{\left\langle t_{n}\right\rangle}{t_{n}^{o}},\frac{t_{s}}{t_{s}^{o}},\frac{t_{t}}{t_{t}^{o}}\right\}$ (17)

where *t*_n_, *t*_s_, and *t*_t_ represent the peak values of the nominal stress when the deformation is either purely normal to the interface or purely in the first or the second shear direction, respectively.

When *f* =1, the cohesive elements transition into the damage evolution stage, leading to a degradation in material stiffness. Damage evolution during the softening phase follows a power law, as shown in Equation (18). In the mixed mode, the power exponent α is set to 1, which refers to the critical fracture energies required to cause failure in the normal, the first, and the second shear directions, respectively.

$\left\{\frac{G_{n}}{G_{n}^{C}}\right\}^{\alpha}+\left\{\frac{G_{s}}{G_{s}^{C}}\right\}^{\alpha}+\left\{\frac{G_{t}}{G_{t}^{C}}\right\}^{\alpha}=1$ (18)

**Macroscale Modeling.** The LS-DYNA software was adopted to develop the 3D macroscale model of our developed LBCs. Three main steps was conducted to perform the modelling process, that is, the generation of ordered flakes models in a spatial domain, the meshing process of the prescribed sample domain, and the 3D mapping process of flakes models into the sample domain. The details of the abovementioned modelling steps was introduced as followings. First, based on the 3D flakes model with random shape and size characteristics reported in our latest study [6], the 3D flakes with large aspect ratio and obvious angular characteristics can be generated, as depicted in **Fig. S4a**. Using the random “Take and Place” algorithm, the 3D flakes model can be randomly delivered in a spatial domain without interacting and overlapping phenomenon. Further, according to the 3D mapping meshing method, the random particle models were meshed using the hexahedron elements. Additionally, based on the position relationship between flakes phase and organic matrix phase (**Fig. S4b**), the material identification algorithm was developed to construct the 3D macroscale model of the LBCs, as shown in **Fig. S4c**. Considering the tiny interfaces between flakes and matrix that was observed in the present experiments, the interfacial transitional zone (ITZ) between these two phases were assumed to be zero, and simulated as a bonding contact.


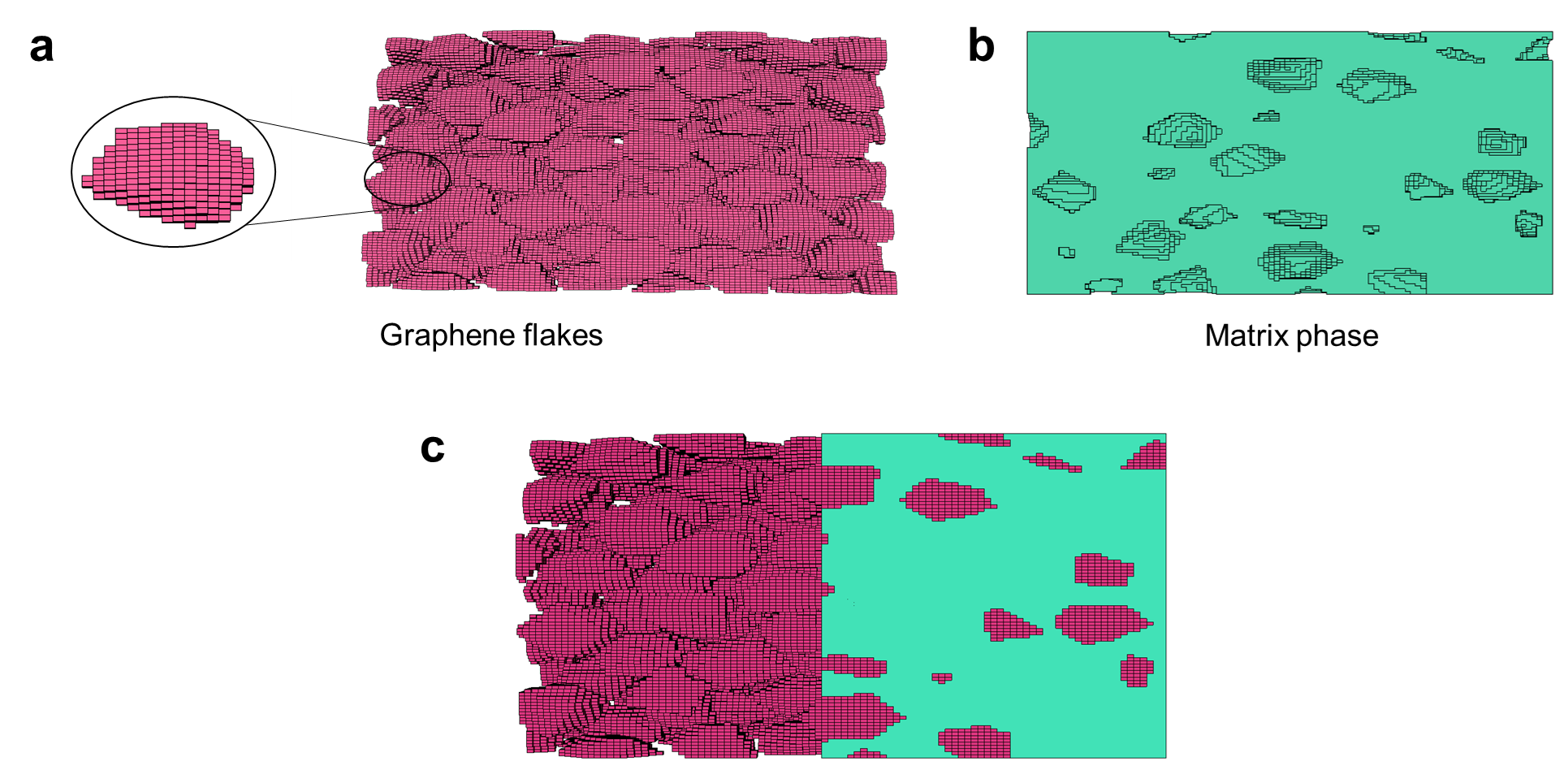


**Fig. S4 Macroscale FE simulation model:** **a**. 3D graphene flakes. **b**. Matrix phase. **c**. LBCs.

**Calculating**. To investigate the flexural behaviors and damage mechanisms of the developed composites, three-point flexural simulations were conducted using the above-established macroscale models. According to the experimental details, the average fraction of the flake model was set 35%. The *MAT_COMPOSITE_DAMAGE material model in LS-DYNA was employed to simulate the material behavior of organic matrix, and the *MAT_ELASTIC material model was selected to simulate the material behavior of graphene flake phase. The steel rod was assumed to be a rigid body without deformation. According to the basic physical and mechanical properties, the critical model parameters of different components, such as mass density ρ, Young’s modulus E , Poisson’s ratio μ, tensile strength f_t_, and compressive strength fc, have been determined and listed in **Table S3**. **Figure S5** shows the notched 3D macroscale models of LBCs subjected three-point flexural loads. Not adding 3D flakes to the model represents a model where the interface does not have a hard phase anchor. According to the experimental program information, the loading rate was set to 0.05 mm/min in simulations.

**Table S3** Materials model parameters of different macroscale components.

| Components | Material model | Key material parameter |
| --- | --- | --- |
| Organic matrix phase | *MAT_COMPOSITE_DAMAGE model | *ρ*=0.55g/ cm^3^ |
|  |  | *E*=3.5 GPa |
|  |  | *μ*=0.36 |
|  |  | *f*_c_=45 MPa |
|  |  | *f*_t_=48 MPa |
| Flakes phase | *MAT_ELASTIC | *ρ*= 2.267g/ cm^3^ |
|  |  | *E*=1.02 GPa |
|  |  | *μ*= 0.16 |
|  |  | *f*_t_= 120 GPa |


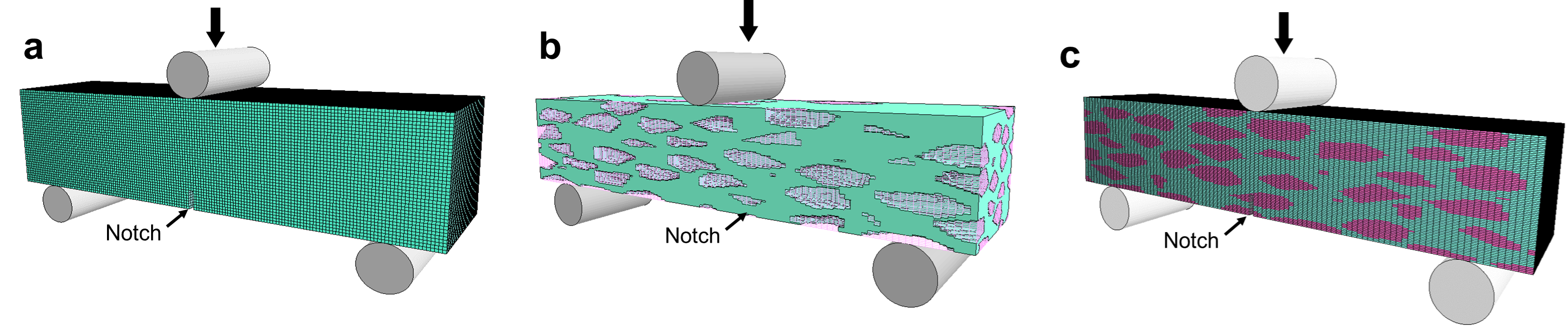


**Fig. S5** **3D FE model of samples subjected to three-point flexural load:** **a.** Notched sample of PVA-PEG-GNs; **b**. Notched sample of PVA-PEG-Hyphae composites; **c**. Notched LBCs sample.


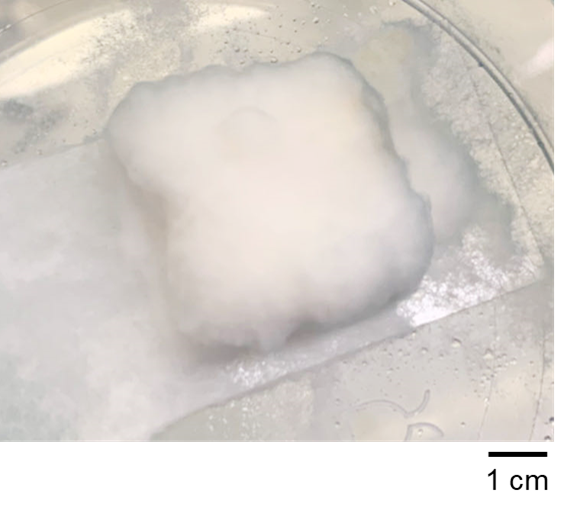


**Fig. S6** The digital photo of *Schizophyllum commune* mycelium.


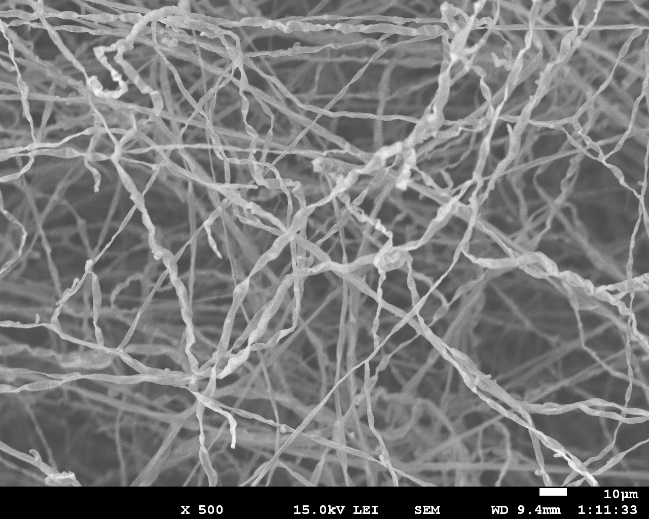


**Fig. S7** SEM image of *Schizophyllum commune* mycelium.


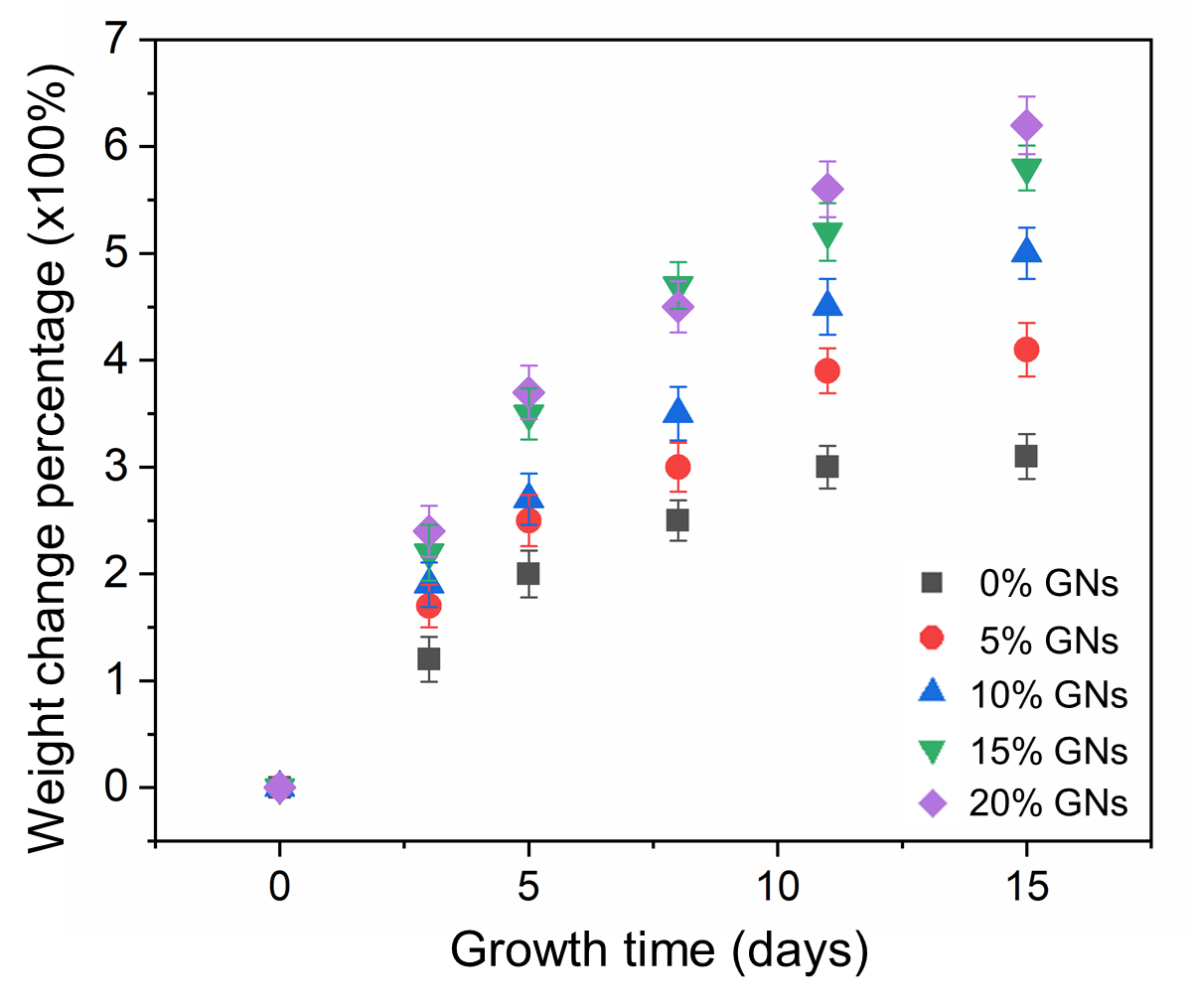


**Fig. S8** Relationship between mycelium growth time and weight change of the obtained composites.


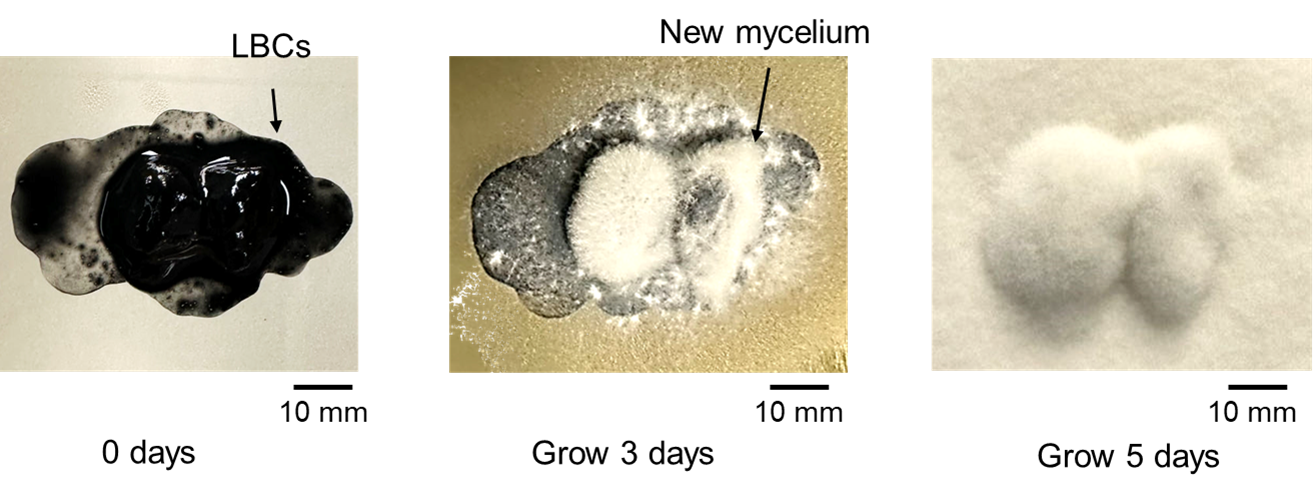


**Fig.S9** Digital camera photos of LBCs before compression and drying process left at room temperature for 3, 5 days.


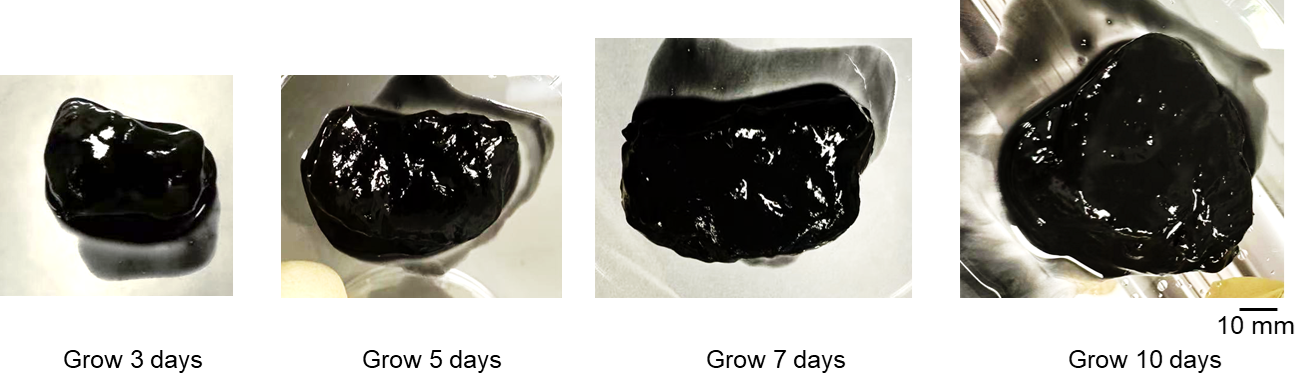


**Fig.S10** Digital camera pictures of the self-regenerative LBCs.


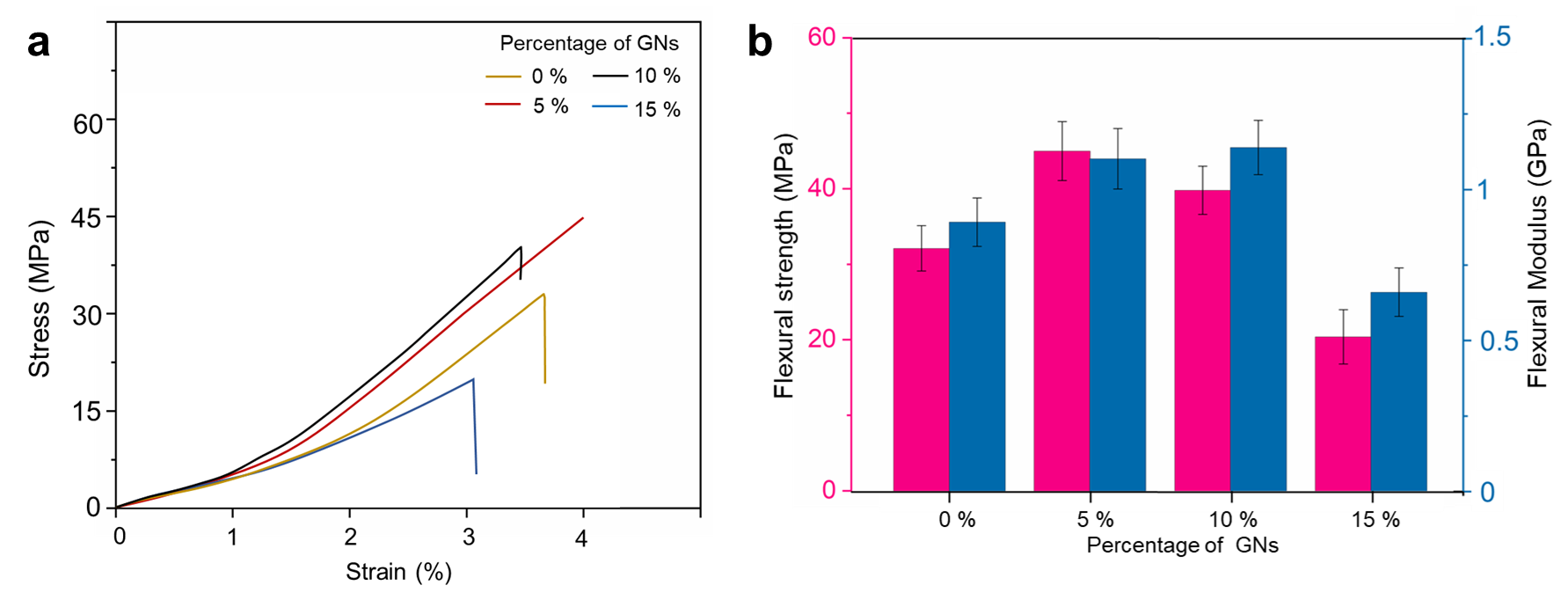


**Fig. S11** Mechanical properties of PVA-PEG-GNs composites: **a**.Typical three-point bending stress-strain curve. **b**. Three-point bending strength and modulus.


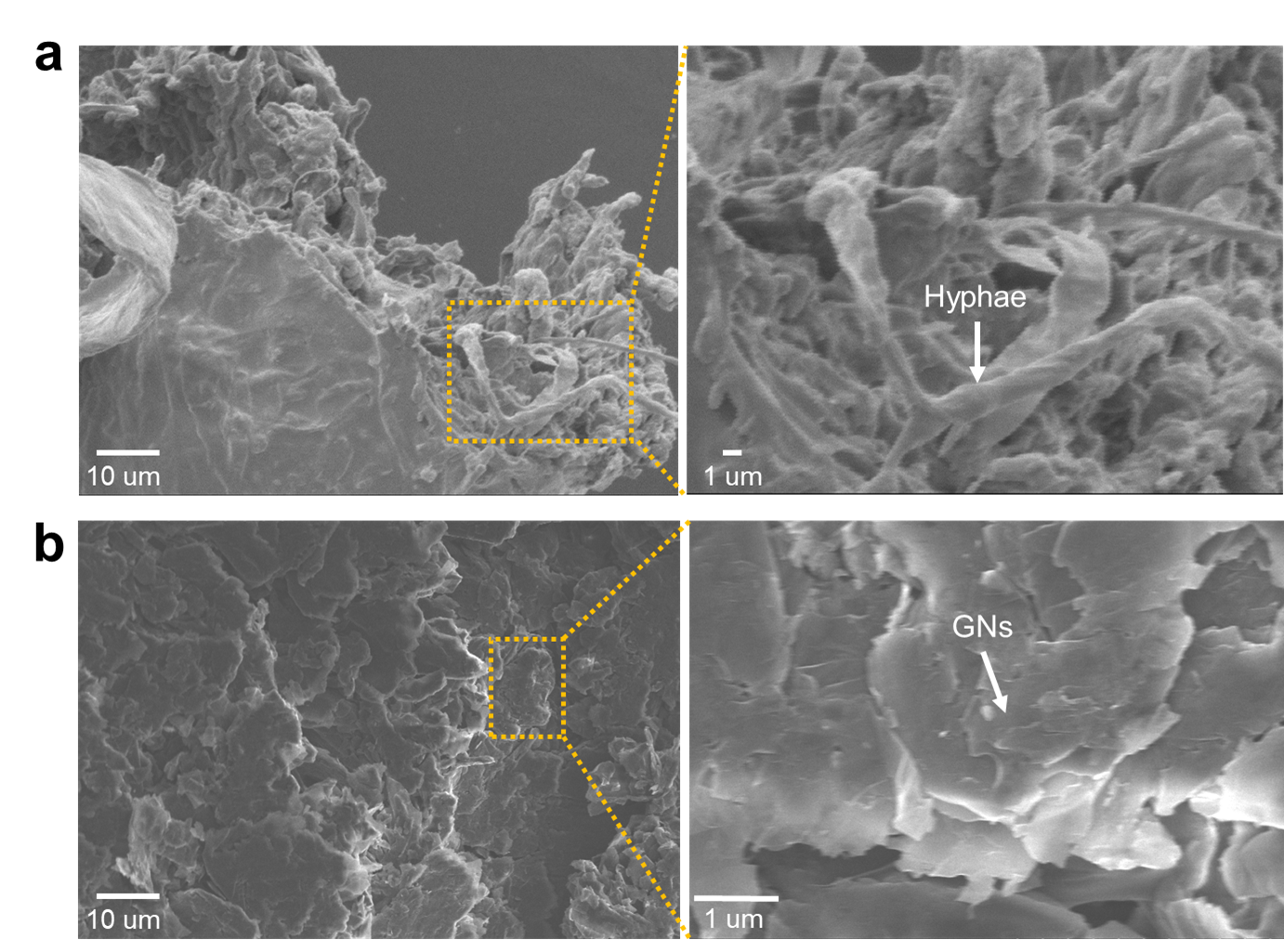


**Fig. S12** SEM images of fracture morphologies after single-edge notched three-point bending test: **a**. PVA-PEG-Hyphae composites. **b**. PVA-PEG-GN composites.


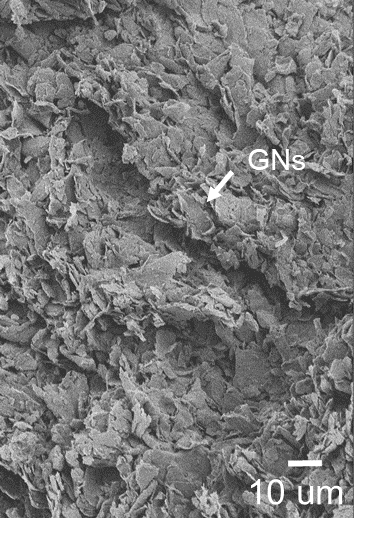


**Fig. S13** SEM image of the fracture morphology of the LBCs after single-edge notched three-point bending test.


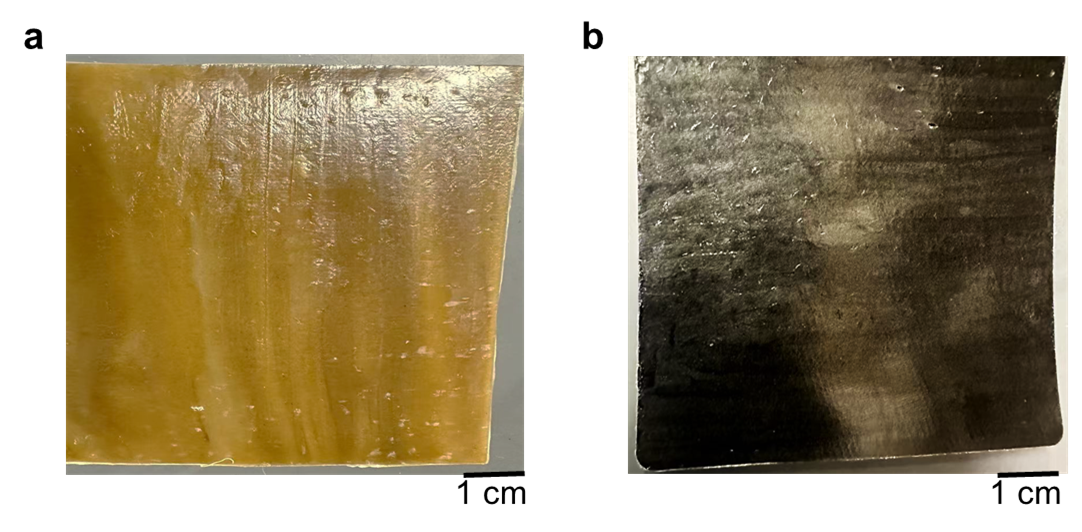


**Fig. S14** Digital camera photos: **a.** PVA-PEG-Hyphae composites. **b.** PVA-PEG-GN composites.

**Table S4** Comparison of mechanical properties of the LBCs with reported biomimetic hierarchical layered structural materials.

| Layered structural materials | Density (g/cm^3^) | Strength (MPa) | Specific strength  (MPa/  Mg/m^3^) | Toughness  (MPa m^1/2^) | Specific toughness  (MPa m^1/2^/ Mg/m^3^) | References |
| --- | --- | --- | --- | --- | --- | --- |
| Bionic mineral-polymer  composites | 1.1 | 83.6±9.5 | 76±8.7 | 2.89±0.33 | 2.63±0.3 | [7] |
| Natural *Cristaria*  *Plicata* nacre | 2.58 | 171.7±51 | 66.55 | 5.9±0.85 | 2.29 MPa | [8, 9] |
| CaCO_3_-based  artificial nacre | 1.54-2.18 |  | 8.26-28.3 |  | 0.69-0.73 | [10, 11, 13] |
| Al_2_O_3_-based  artificial nacre | 1.61-1.83 |  | 19.1-72.73 |  | 1.56-2.8 | [11] |
| Graphene printed nacre | 1.04-1.08 |  | 30 |  | 2.4 | [12] |
| Wooden artificial nacre | 1.59 ± 0.05 | 121.13±17.55 | 76.18±11.04 | 11.77±1.27 | 7.40±0.80 | [13] |
| LBCs | 0.89 | 82.6 | 92.8 | 6.5 | 7.3 |  |

**Table S5** Electrical conductivity of the LBCs.

| LBCs | Size  （l $\times$ w $\times$ t）(m） | Density  （g/cm^3^） | Resistance （Ω） | Electrical conductivity  (S/m) |
| --- | --- | --- | --- | --- |
| Along the alignment direction | 0.00668 $\times$ 0.00642 $\times$ 0.00051 | 0.89±0.2 | 484±54 | 254.482±8.5 |
| Vertical the alignment direction | 0.00668 $\times$ 0.00642 $\times$ 0.00051 | 0.89±0.2 | 1700±142 | 0.0245±0.05 |

**Table S6** Density and electrical properties of the PVA-PEG-GN composites prepared by blending methods.

| PVA-PEG-GN composites | Size  （l $\times$ w $\times$ t）(m） | Density  （g/cm^3^） | Resistance （MΩ） | Electrical conductivity  (S/m) |
| --- | --- | --- | --- | --- |
| Overall | 0.055 $\times$ 0.038 $\times$ 0.0032 | 1.21±0.2 | 0.5 | 0.0001 |

**Code for visualizing resistance change in computer**

Serial myPort; //

color touchColor = color(255, 0, 0); //

color defaultColor = color(0); //

void setup() {

size(400, 400);

//

String portName = "COM3"; //

myPort = new Serial(this, portName, 9600);

}

void draw() {

background(255); //

//

while (myPort.available() > 0) {

String data = myPort.readStringUntil('\n');

if (data != null) {

//

int touchState = Integer.parseInt(data.trim());

//

fill(touchState == 10 ? touchColor : defaultColor);

}

}

//

drawExclamation(width / 2, height / 2, 240); //

}

void drawExclamation(float x, float y, float size) {

float lineLength = size * 0.6;

float lineWidth = size * 0.1;

float dotSize = size * 0.2;

//

rectMode(CENTER);

rect(x, y - size * 0.25, lineWidth, lineLength);

//

ellipse(x, y + size * 0.25, dotSize, dotSize);

}

void keyPressed() {

// myPort.write("10\n");

}

**Vedio S1**. Self-monitoring of finger-applied pressure loading.

It shows that when a load is applied by a finger, the load can be converted into a signal change on the computer.

**Vedio S2**. Self-monitoring visualization of finger-applied pressure loading.

It demonstrates that when a load is applied by a finger, the load can be converted into a computer visualization, with the black hearts on the computer screen turning into green.

**Vedio S3**. Self-monitoring visualization of steel ball impact loading.

It shows that when the LBCs are hit by free-falling steel balls, the impact force can be transformed into a computer visualization, with the black exclamation mark on the computer screen turning into red.

**References**

1. H. W. Zhao, et al., *Science* **375**, 551–556 (2022).
2. E. Roumeli, R. Hendrickx, L. Bonanomi, A. Vashisth, K. Rinaldi, C. Daraio, *PNAS* **119** (15), e2119523119 (2022).
3. S. F. Zhou, T. Khan, K. Jin, et al., *Adv. Funct. Mater.* **32** (14), 2109881 (2021).
4. H. Wang, J. Tao, Z. Y. Wu, et al., *Adv. Sci.* 2309370 (2024).
5. P. Tran, T. D. Ngo, A. Ghazlan, et al., *Compos. Part B: Eng.* **108**, 210–223 (2017).
6. I. Yungerman, I. Starodumov, A. Fulati, et al., *J. Phys. Chem. B* **126**, 3961–3972 (2022).
7. A. Xin, Y. Su, S. Feng, M. Yan, et al., *Adv. Mater.* **33**, 2006946 (2021).
8. L. B. Mao, et al., *Science* **354**, 107–110 (2016).
9. H. L. Gao, S. M. Chen, L. B. Mao, Z. Q. Song, H. B. Yao, H. Cölfen, X. S. Luo, F. Zhang, Z. Pan, Y. F. Meng, et al., *Nat. Commun.* **8**, 287 (2017).
10. H. K. Raut, A. F. Schwartzman, R. Das, F. Liu, L. Wang, C. A. Ross, J. G. Fernandez, *ACS Nano* **14**, 9771 (2020).
11. S. M. Chen, H. L. Gao, X. H. Sun, Z. Y. Ma, T. Ma, J. Xia, Y. B. Zhu, R. Zhao, H. B. Yao, H. A. Wu, S. H. Yu, *Matter* **1**, 412 (2019).
12. Y. Yang, et al., *Sci. Adv.* **5**, eaau9490 (2019).
13. Z. Qiu, L. Z. Lang, Z. Y. Yu, et al., *Adv. Funct. Mater.* **34**, 2310096 (2024).
